# Molecular fusion of surfactant and Lewis-acid properties for attacking dirt by catalytic bond cleavage

**DOI:** 10.1038/s41598-021-84654-3

**Published:** 2021-03-04

**Authors:** Marvin L. Frisch, Sebastian Polarz

**Affiliations:** 1 Institute for Inorganic Chemistry, Leibniz-University Hannover, Callinstrasse 9, 30167 Hannover, Germany; 2 Department of Chemistry, University of Konstanz, Universitaetsstrasse 10, 78457 Konstanz, Germany; 3 Institute of Chemistry, Technical University Berlin, Strasse des 17. Juni 124, 10623 Berlin, Germany

**Keywords:** Catalysis, Coordination chemistry, Environmental chemistry, Materials chemistry, Nanoscale materials, Soft materials

## Abstract

The capability of ordinary surfactants in solubilizing hydrophobic compounds can come to a limit, if the extension of a contaminant is too large. An attractive goal is the development of surfactants which can actively reduce the size of dirt. Because strong Lewis acids are known to catalyze both bond formation and cleavage, an integration into the surfactant's molecular framework is tempting. End-group functionalized hepta-dentate ligands, which coordinate to metal ions preventing deactivation by hydrolysis over a broad range of pH values while maintaining strong Lewis-acidity, are herein presented. After proof of amphiphilicity and surfactant characteristics, catalytic properties are investigated for different reactions including the cleavage of proteins. The compounds perform better than benchmark catalysts concerning the attack of unreactive amide bonds. A study with two Sc^3+^ species as the active site, one non-amphiphilic, the other one being surface-active, underlines the positive effect of surfactant properties for boosting catalytic efficiency.

## Introduction

The main function of surfactants is amphiphilicity, meaning they are able to decrease the energy of interfaces. Interfacial activity is the key for numerous applications in research, industry and in everyday life. Surfactants are the crucial constituent in almost all detergents on the market. However, classical surfactants reach their limit when the extension of the contaminants is too large. Then, mechanical abrasion is a possibility for their removal. Cleaning by scrubbing can be difficult depending on the accessibility of the polluted surface, on its geometry, on its mechanical stability and many other factors. Another well-known approach is the use of additives, e. g. oxidation agents, in detergents. These additives are unspecific and may accelerate unwanted wear down of materials. Unfortunately, oxidants are often not successful for many organic contaminants on cookware resulting from the Maillard reaction, for instance. As a major drawback, dilution as a consequence of dissolution requires the use of a significant excess, when working with two different compounds separately. The collaboration of the two components, surfactant and additive, occurs by chance.

Thus, the implementation of an active entity capable of attacking dirt on a molecular level into a surfactant is a different concept because the simultaneous impact of both functionalities is programmed in the molecular framework. The implementation of a Lewis-acid into a surfactant would open up multiple possibilities beyond cleaning applications, e. g. in phase-transfer catalysis. Next generation surfactants possess additional properties beyond amphiphilicity^1^. In particular, the combination with catalytic properties has raised large interest. An important review has been published by Scarso et al*.* in 2019^2^. There are only a few reports on metal species or catalysts as an integral part of the surfactant's molecular architecture, for instance as a head group. This situation has not changed a lot since first original papers and reports on metallosurfactants have been published^3–6^.

A relevant catalytic mechanism relies on the activity of a strong Lewis acid in lowering the activation barrier for bond cleavage and bond formation^7^. Powerful Lewis acids are known from solid-state chemistry used in heterogeneous catalysts^8–10^. For the combination with a surfactant, one obviously seeks for homogeneous conditions and molecular type catalysts^11^, such as compounds containing metal atoms (e. g. Zn, Sc, Ti, Zr, Yb) preferentially in their highest oxidation state. However, the resulting electrophilic character leads to a problem in aqueous solution^12^. In alkaline conditions, in particular, the formation and precipitation of the corresponding metal hydroxides and oxy-hydroxides leads to an irreversible deactivation of the catalyst. There has been substantial effort regarding the development of molecular Lewis-acid catalysts for an application in aqueous systems as outlined by Kobayashi in several seminal articles^11,13^. The most promising route for the stabilization of the metal centre is the coordination to multi-dentate ligands. Importantly, the electrophilicity has to remain sufficiently high. Beyond that, free sites at the metal centre for substrate coordination are still required.

**Figure 1:**
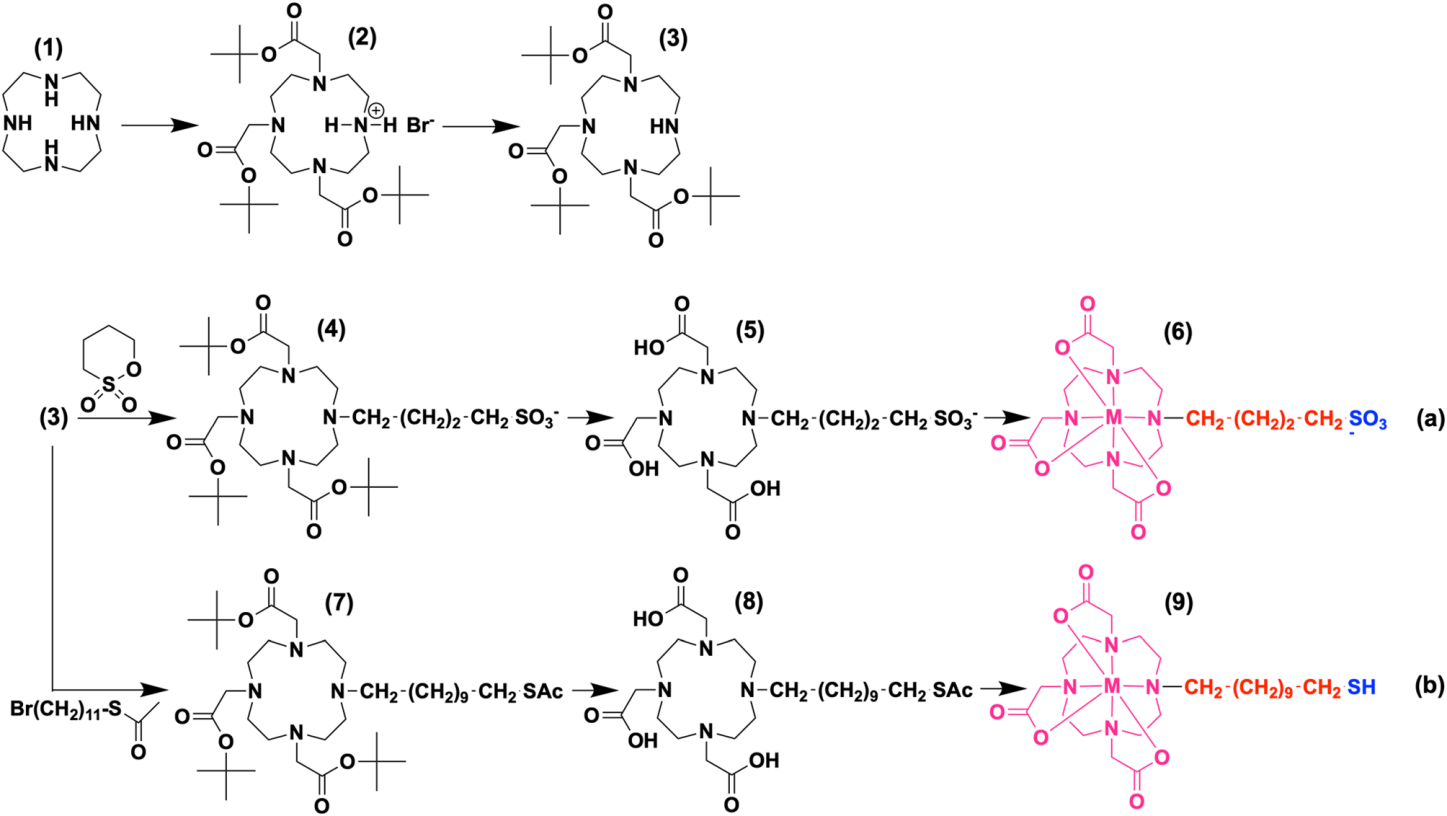
Synthesis routes to new surfactants with DO3A-metal complexes as constituents. (**a**) sulfonic acid terminated bolaform compounds M-DO3A-C_4_-SO_3_. (**b**) thiol terminated bolaform compound M-DO3A-C_11_-SH. Blue = hydrophilic; red = hydrophobic; purple = predominantly hydrophobic. M^3+^  = Ce^3+^(i), Sc^3+^(ii), Yb^3+^(iii).

Our goal are surfactants containing an active Lewis acid as a head group, which is stable even under alkaline conditions in aqueous media. Our group has presented novel amphiphilic compounds comprising a cyclen derivative modified by three acetic acid groups (DO3A) to which one alkyl group with adjustable chain length is attached (C_n_)^14,15^. We were interested in the special magnetic properties of DO3A-C_n_ complexes with metals such as M = Mn^2+^, Co^2+^, Ni^2+^, Dy^3+^. Lewis-acidic metals have not been used and catalytic properties have not been studied so far. Here, the ligand system is developed further and strong Lewis acids such as Yb^3+^, Ce^3+^ or Sc^3+^ (see Fig. 1) are coordinated, followed by a study of their amphiphilic properties and testing of their catalytic activities.

## Results

The charge of the deprotonated ligand DO3A-C_n_ is (3-)^14,15^. In combination with a metal cation M^3+^, the charge reduces to (0), so does the solubility in water. As M^3+^ (Yb^3+^, Ce^3+^, Sc^3+^) are preferred due to their enhanced electrophilicity compared to weaker Lewis acids (e. g. Zn^2+^), the introduction of an additional polar group becomes necessary for providing sufficient solubility in aqueous media. Surfactants with two head-groups on opposing sides of the hydrophobic chain are also called bolaform surfactants^16,17^.

### ***Bola M-DO3A-C***_***n***_***-SO***_***3***_*** compounds***

Sulfonic acid groups seem promising (Fig. 1) because of the low pK_a_ value ( ≈ -7) of (-R-SO_3_H). Based on the experiences described above^14,15^, a viable conclusion is that a zero-charged M^3+^-DO3A^3-^ entity is at least partially hydrophobic. If M^3+^-DO3A^3-^ adds to the hydrophobic block of a surfactant, a relatively short alkyl chain as a spacer to the sulfonic groups should be sufficient to evoke amphiphilic properties. Thus, DO3A-C_4_-SO_3_ (**5**) represents our first target compound (Fig. 1a). Further details about the synthetic steps are given in experimental part^18^. The most important step is reaction of the free base (**3**) in a ring-opening nucleophilic substitution with 1,4-butane sultone followed by the acid-catalysed deprotection. Characterization of the resulting organic ligand (**5**) was accomplished via NMR spectroscopy and mass spectrometry analysis, which are given in the Supporting Information in SI-1 + 2.

**Figure 2:**
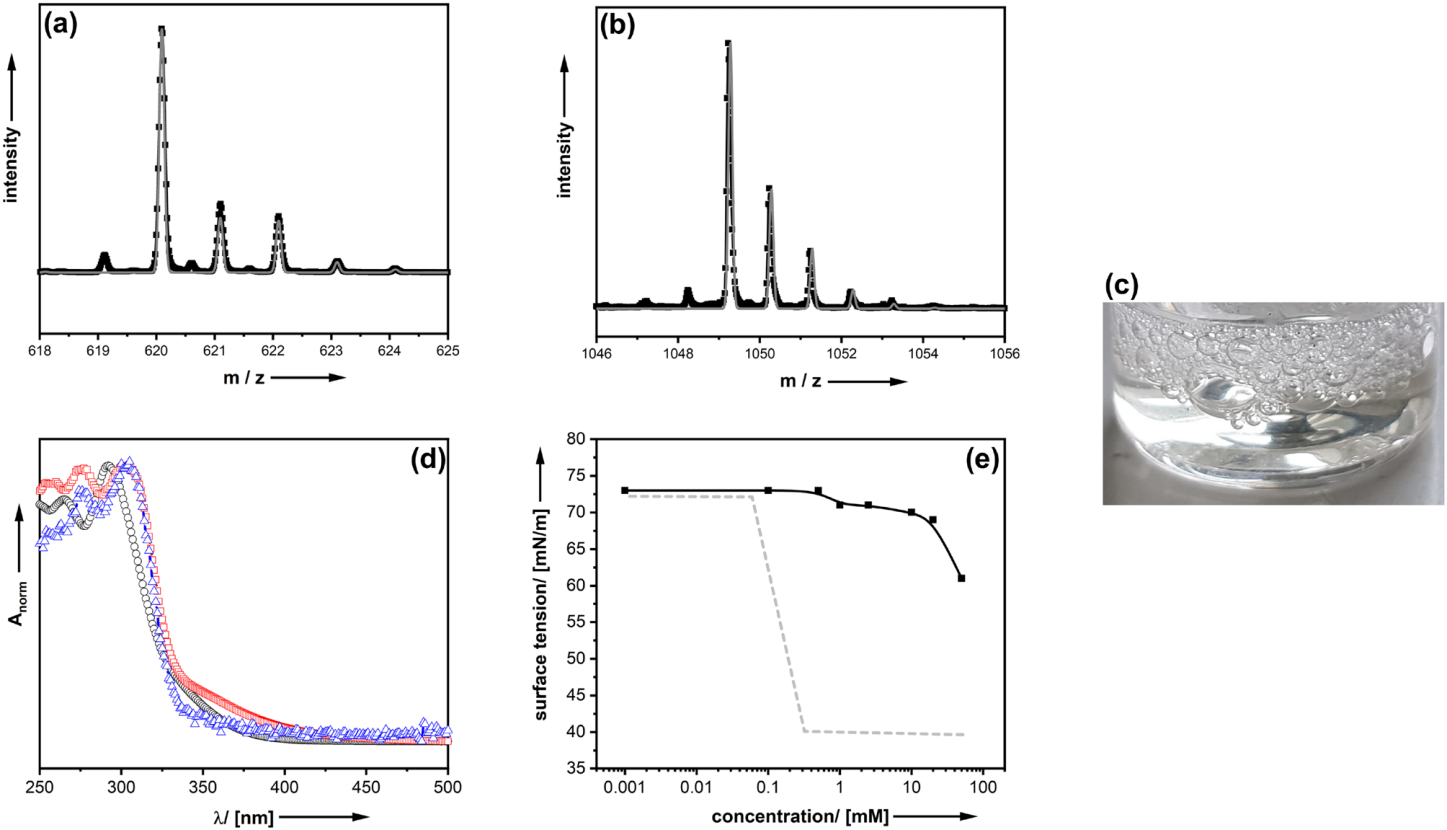
Characterization and hydrolytic stability tests of representative M-DO3A-C_4_-SO_3_ compounds dissolved in aqueous solution at neutral and alkaline pH values. (**a**) ESI–MS pattern of Ce-DO3A-C_4_-SO_3_ (black = measurement; grey = simulation for M = [Ce-DO3A-C_4_-SO_3_H]H^+^. (**b**) ESI–MS pattern of Sc-DO3A-C_4_-SO_3_ (black = measurement; grey = simulation for M = [Sc-DO3A-C_4_-SO_3_]_2_. (**c**) Photographic image of a solution of Ce-DO3A-C_4_-SO_3_ in water. (**d**) UV–VIS spectra (absorption normalized) of Ce-DO3A-C_4_-SO_3_ in water at pH = 7 (black circles), pH = 12 (red squares) and pH = 12; t = 48 h (blue triangles). (**e**) concentration dependent surface tension for Ce-DO3A-C_4_-SO_3_; black = experimental points, grey = idealized behavior for a surfactant as a reference.

The final coordination of the metal centres (**5**) → (**6**) was proven by a combination of methods, which are summarized in SI-3. Electrospray ionization mass spectrometry (ESI–MS) is a very efficient technique for the characterization of metallosurfactants, as the metal often contributes a characteristic isotope pattern. Figure 2a,b shows two representative ESI–MS patterns for Ce-DO3A-C_4_-SO_3_ and Sc-DO3A-C_4_-SO_3_. For the diamagnetic complexes, e. g. Sc-DO3A-C_4_-SO_3_, NMR spectroscopy was feasible as an additional characterization technique (see Supporting Information Fig. SI-3). Attenuated total reflection infrared spectroscopy (ATR-IR spectroscopy) confirms a strong coordination of the different Lewis-acidic metal ions. Sc^3+^ was selected, because Kanai et al*.* have presented a study in 2017 about triflate salts used for peptide bond-cleavage^19^. However, the authors have not used amphiphilic compounds nor demonstrated stability of the catalysts at elevated pH values in aqueous medium. An advantage of Ce-DO3A-C_4_-SO_3_ is that it is very simple to decide whether treatment of the compound with water induces a release of the metal atom in particular at high pH values (pH 12). The addition of hydroxide ions to a solution of free Ce^3+^ ions induces a rapid formation of a purple precipitate corresponding to a Ce(III)-oxide-hydroxide phase^20,21^. Visual inspection (Fig. 2c) indicates that there is no precipitate for Ce-DO3A-C_4_-SO_3_, and the solution still looks clear. Therefore, it seems Ce^3+^ is sufficiently stabilized against hydrolysis. The same conclusion can be drawn from UV–VIS spectra shown in Fig. 2d. Ce-DO3A-C_4_-SO_3_ shows good solubility in water, and at neutral pH one obtains a colourless solution with absorption bands in the UV-region (λ_max_ = 295 nm). The absorption pattern does not change at pH 12. There is only a slight red-shift of the bands (λ_max_ = 302 nm), which can be explained by an altered ionic strength of the solution. There are no indications for a hydrolytic attack of hydroxide ions even after prolonged times (pH 12, t = 48 h). The compound is stable in water. Analogous experiments were performed with Sc-DO3A-C_4_-SO_3_ and the same result was obtained.

The conclusion that there is not leaching of the metal ions is in perfect agreement with literature on the quantitative determination of complex formation constants K of the DOTA ligand with diverse metal cations. log(K) is typically in the range 15–20 for lanthanides^22^, and can even reach 27 for Sc^3+^^23^. This explains, why we have not seen a change in the UV–VIS signals over time and also no precipitation of (oxy-)hydroxides.

A characteristic shape of the concentration-dependent surface tension is expected for a compound with surfactant properties, *i. e.* a steep decrease due to the population of the air–water interface by surfactant molecules, followed by a plateau, when the surface is fully covered and aggregates (e. g. micelles) form in solution. Figure 2e illustrates the behaviour of Ce-DO3A-C_4_-SO_3_ is rather different, as the surface tension of the aqueous phase remains almost unchanged up to very high concentrations of the compound. Consequently, Ce-DO3A-C_4_-SO_3_ is not or only weakly surface-active. Even though all carboxylate groups are coordinated (Fig. 1/(6)), the Ce-DO3A moiety is not hydrophobic enough to complement the short C_4_ alkyl chain. The same result was obtained for all M-DO3A-C_4_-SO_3_.

### ***Bola M-DO3A-C***_***11***_***-SH compounds***

To improve the amphiphilic character, the hydrophobic block of the compounds has to be enlarged. Beyond that, the polarity of the second terminal functionality was adjusted, *i. e.* a less polar thiol group was chosen, which still provides sufficient solubility in aqueous media. Therefore, for the introduction of a longer alkyl chain consisting of eleven –CH_2_ units, a nucleophilic substitution reaction with a thioacetate-terminated C_11_ alkyl bromide was chosen (Fig. 1b). In a subsequent step, the acetate group can be cleaved affording a thiol group. The characterization of the thioacetate-protected compound (**8**) is summarized in Fig. SI-4.

**Figure 3:**
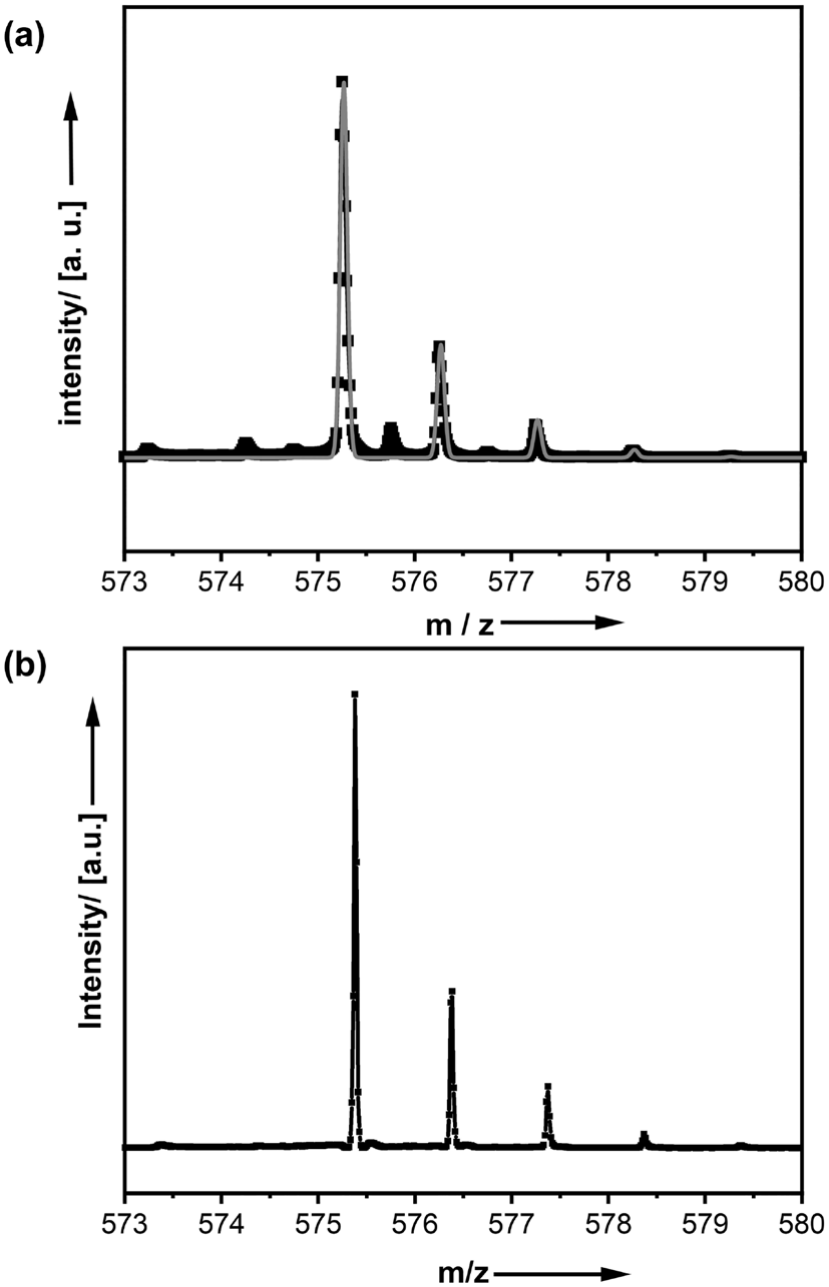
Characterization of Sc-DO3A-C_11_-SH surfactant synthesized according to Fig. 1b. (**a**) ESI–MS pattern of Sc-DO3A-C_11_-SH; black = experimental pattern, grey = pattern simulated for M = [Sc-DO3A-C_11_-SH]H^+^. (**b**) ESI–MS pattern after 48 h at pH 12.

The coordination of metal ions was found to be highly favoured under alkaline conditions. The ESI–MS pattern of Sc-DO3A-C_11_-SH is shown in Fig. 3a, proving the successful preparation of the compound. Additional characterization via NMR and ATR-IR can be found in Fig. SI-5. In analogy to the sulfonate-terminated C_4_ compounds discussed above, hydrolysis is strongly retarded for Sc-DO3A-C_11_-SH. Even after days at high pH values of 12, there is almost no precipitation occurring (see SI-6 in the Supporting Information for further details). Further evidence for the high stability against hydrolysis is provided by ESI–MS analysis after 48 h at pH 12 (Fig. 3b), indicating that the compound is still present in the solution and has not undergone significant decomposition via hydrolysis.

**Figure 4:**
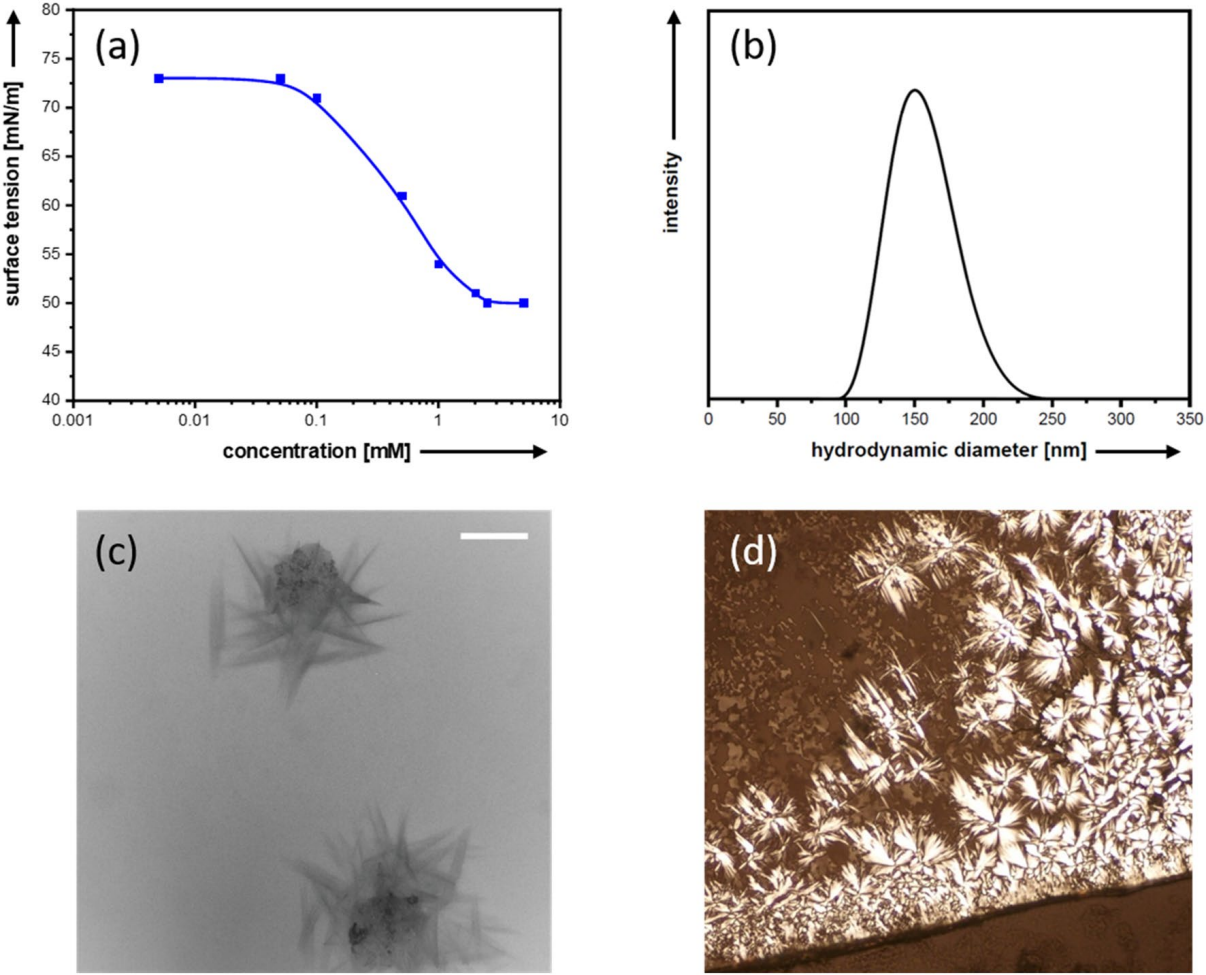
Surfactant-like behavior of Sc-DO3A-C_11_-SH in water. Concentration dependent surface tension (**a**), DLS size distribution function (**b**) representative TEM image of a dried solution (**c**; scale bar = 100 nm), microscopy image of a high concentration phase under crossed polarizers (**d**).

M-DO3A-C_11_-SH shows significantly improved amphiphilic properties. The findings are discussed for Sc-DO3A-C_11_-SH as a representative case. As first indication for surface activity, strong foaming was observed after shaking an aqueous solution. More importantly, concentration-dependent tensiometry experiments reveals surface-activity (Fig. 4a). The critical aggregation concentration is at 2.5 mM, which lies in between that of classical surfactants such as sodium dodecyl sulfate (SDS; 8.2 mM) or polyoxyethylene-akyl surfactants (≈ 0.3 mM). Above the cmc, aggregates are formed in solution. According to dynamic light scattering (DLS) measurements (see Fig. 4b), the size of these aggregates is about 150 nm on average, which strongly suggests the presence of larger aggregates such as vesicles or lamellar structures. As the metal-complex is rather bulky, and our experiments have shown it should be treated as a second head group, it is not surprising that the compound behaves different from classical surfactants. To confirm our hypothesis, transmission electron microscopy (TEM) was used (Fig. 4c). The micrographs show aggregated furled sheets. The size of those particles fits to the DLS results and indicate a tendency of the surfactant to form lamellar structures. As shown in SI-7 in the Supporting Information, multi-layered sheet-like structures can also be found. At higher concentration, surfactants can form lyotropic liquid crystals (LLCs). A polarization microscopy image of such a phase is shown in Fig. 4d. The birefringence clearly indicates the presence of an optically anisotropic phase. The observed pattern (e. g. occurrence of Maltese crosses)^24^ fits to the presence of a lamellar phase.

### Catalytic testing

As already mentioned, it is a well-known fact that the coordination by a multi-dentate ligand negatively affects the degree of electrophilicity of a metal ion centre and, hence, also its Lewis acidity. The question arises whether there is any catalytic activity remaining despite the coordinative saturation of the metal centre. It is common in literature that Lewis-acid catalysis is investigated by bond formation reactions. For instance, a Mukaiyama aldol-type reaction was already used by Kobayashi et al*.* as a test for Lewis-acid catalysis^25^. The authors reported high activities for d- and f-block metal triflates when either a co-solvent, e. g. THF, or a surfactant, e. g. SDS, is added to the aqueous reaction mixture.

Thus, a Mukaiyama aldol reaction and a Michael addition were chosen as representatives for C–C bond formation reaction. Notably, despite the strong coordination by hepta-dentate ligands, Sc-DO3A-C_11_-SH shows high catalytic activities comparable to reported Lewis-Acid-surfactant-combined catalysts (LASCs) such as dodecyl sulfate and Sc(III) or Yb(III) as counter-ion^26^. The more polar compound Sc-DO3A-C_4_-SO_3_ performs significantly inferior affording no reaction products at all (*cf.* Table SI-1 in the Supporting Information), which can be explained by its weaker amphiphilic properties compared to Sc-DO3A-C_11_-SH. With a conversion of 30%, the latter reaches a similar activity as literature-reported LASCs but is better protected against deactivation by unwanted precipitation at higher pH values. The broad applicability of M-DO3A-C_11_-SH is confirmed by the results on the Michael addition with indole as hydrophobic, water-immiscible reactant. Again, the surfactant catalyst Sc-DO3A-C_11_-SH outperforms the non-amphiphilic Sc-DO3A-C_4_-SO_3_ with 26% conversion for only 1 mol-% of catalyst compared to 11% conversion for a ten-fold higher catalyst concentration, respectively (*cf.* Table SI-2 in the Supporting Information). There was no indication of a side-reaction between the thiol group –SH with any of the electrophilic reagents in the test reactions. Unless strong bases are added to the reaction mixtures, the –SH group remains stable and no deprotonation occurs.

Bond fission is more relevant for the catalytic attack of organic contaminants. The hydrolytic cleavage of gly-ser dipeptides and the fragmentation of a model protein were selected (Table 1). The conversions were determined in analogy to the procedure reported in reference papers by quantification of ^1^H-NMR spectra^27,28^. Reference experiments were performed using the bare organic ligand for ensuring catalytic activity stems from the presence of the metal cation. Furthermore, the integrity of the surfactant-like compounds was verified by analysis (e. g. ESI–MS) after the reaction (see SI-8). Studies were performed with the Sc-containing compounds (**6ii**) and (**9ii**), which are both stable against hydrolysis even under strong basic conditions. Because the catalytically active entity is identical, it will be interesting to see, whether the differences in amphiphilic character and polarity again have an effect on the catalytic performance in the cleavage of un-activated amide bonds^29^. The compounds developed in this work show high activity (*cf.* Table SI-3 in the Supporting Information). Although the dipeptide is polar and well soluble in water, the amphiphilic character of the catalysts is of importance. The amphiphilic Sc-DO3A-C_11_-SH performs better and is 2.5-times more active, affording conversion degrees of 32%. Remarkably, to the best of our knowledge, the compound ranges among the top performing Lewis-acidic catalysts stated in literature. Ly et al^30^*.* achieved a yield of about 35% of cleaved amino acids glycine and serine using an equimolar amount of catalyst (a dimeric Keggin-type polyoxometalate (POM) containing Zr(IV) ions as active centres), but at acidic conditions, which is known to significantly accelerate the kinetics of a hydrolytic amide bond cleavage^31^. Besides the cleaved amino acids, a minor amount of the cyclic compound c-(gly-ser) was reported by Ly et al*.* Herein, the amount of the latter was more than four times higher compared to their results. From their investigations, it can be assumed that the hydrolytic cleavage of other dipeptides, e. g. gly-gly, or cyclic peptides will also be feasible using the Lewis-acidic metal–organic complexes developed in this work.

**Table 1:** Overview over the obtained conversions for peptide bond fission reactions and comparison to best-performing systems in literature.

Cleavage agent	Molar ratio cleavage agent to reactants	Reaction	Reaction conditions	Conversion
This work (6ii)	1	Dipeptide cleavage (gly-ser)	24 h, 70 °C, pH 7.0	13%
This work (9ii)	1	Dipeptide cleavage (gly-ser)	24 h, 70 °C, pH 7.0	32%
dimeric Zr^(IV)^-POM^30^	1	Dipeptide cleavage (gly-ser)	24 h, 60 °C, pD 5.4 (pH ~ 5.0)	~ 35%
This work (6ii)	~ 1000	BSA cleavage	60 °C, pH 6.0	100% after 16 h
This work (9ii)	~ 1000	BSA cleavage	60 °C, pH 6.0	100% after 16 h
Co^(III)^-cyclen-C15^33^	~ 67	BSA cleavage	50 °C, pH 9.0	100% after 16 h
Cu^(II)^-cyclen-C15^33^	~ 67	BSA cleavage	50 °C, pH 9.0	100% after 39 h
Cu^(II)^-cyclen^33^	~ 20,000	BSA cleavage	50 °C, pH 9.0	~ 0% after 48 h

**Figure 5:**
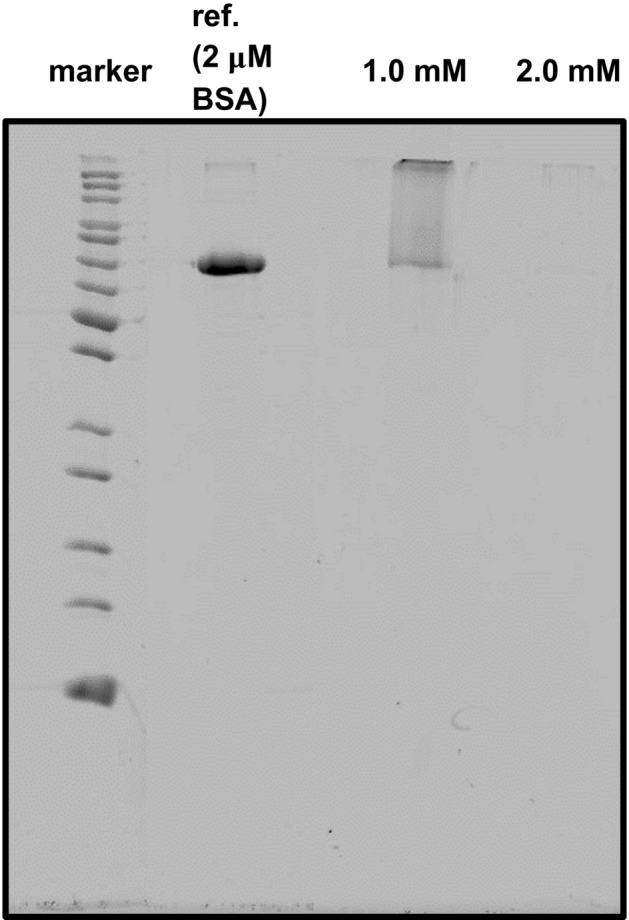
SDS-PAGE of the hydrolytic cleavage of BSA at pH 6 and 60 °C for 16 h using Sc-DO3A-C_11_-SH (**9ii**) as a surfactant-cleavage agent. A reference experiment without cleavage agent using a concentration of 2 µM BSA is shown next to the marker.

Because of its superior performance, we have focussed on Sc-DO3A-C_11_-SH for our final tests. A protein is a much more realistic compound for an organic contaminant present in practical fields of application of Lewis acidic surfactants. Thus, SDS-PAGE was performed using BSA (bovine serum albumin) as a protein (Fig. 5). SDS-PAGE stands for sodium dodecyl sulfate polyacrylamide gel electrophoresis^29^, and it is a well-established method for the analysis of polypeptides and its fragments. One can see, Sc-DO3A-C_11_-SH is active. At neutral pH and at room temperature, the stability of a peptide bond is extraordinarily high with a reported half-life of up to 600 years^32^. Lewis-acidic compounds can significantly accelerate the cleavage reaction kinetics. Indeed, quantitative fragmentation of BSA has taken place after 16 h of treatment in water at pH 6 and a concentration of the cleavage agent of 2.0 mM (1000-fold molar excess). Perera-Bobusch et al*.*^33^ reported excellent activities for Co^3+^ coordinated by a cyclen moiety to which one C_16_ alkyl chain was covalently attached. After 16 h of incubation, a 67-fold molar excess of Lewis acidic cleavage agent respective to BSA was necessary for quantitative cleavage. However, the cleavage reactions were performed at pH 9. When the pH was set to 7.4, significant decreases in cleavage activities were admitted by the authors. Disadvantageously, Co^3+^ species are highly toxic and could easily leach into the surrounding solution, as the complex formation constant is rather low for mono-alkylated cyclen ligands. For a similar system comprised of non-toxic Cu^2+^ ions as active species for bond cleavage, inferior cleavage properties were observed. The high proteolytic activity of Sc-DO3A-C_11_-SH in this study is expected to be a consequence of the long hydrophobic alkyl chain that favours interaction with the hydrophobic pockets in the protein. Besides high activity, sufficient stability of the compound in the presence of the protein at elevated temperature is manifested since there is no formation of a precipitate or gel during the cleavage reaction. Moreover, a pre-treatment in alkaline solution prior to the cleavage reactions had no adverse effect on the cleavage activities of the Lewis-acidic compounds due to their sufficient protection against hydrolysis by the strongly coordinating DO3A ligand (see *4.2.2* in the Supporting Information).

## Conclusion

It occurs very often that the character of dirt is at least partially hydrophobic. Sometimes the skills of conventional surfactants to solubilize such hydrophobic contaminants are not sufficient. Thus, our aim was the exploitation of surfactants, which contain a catalytically active constituent with strong Lewis-acidity. A ligand containing a cyclen derivative modified by three acetic acid groups (DO3A) in a terminal position attached to one alkyl chain was prepared. An additional group, either -SO_3_H or -SH, responsible for higher solubility in water was introduced at the other terminus of the aliphatic alkyl chain. After coordination to different metal ions like Zn^2+^, Ce^3+^, Yb^3+^ and, most importantly, Sc^3+^, depending on the length of the hydrocarbon chain, a non-amphiphilic and a surfactant-like compound were obtained. Both compounds were highly stable in water and survived the treatment at pH 12 for more than two days. It was demonstrated that the metal-DO3A complex is still catalytically active regarding bond-formation and bond-cleavage reactions. Despite the strong electron donating properties of the macrocyclic ligand being essential for hydrolytic stability over a broad range of pH values, the metal ion still remains catalytically active in different Lewis-acid catalyzed bond formation as well as cleavage reactions. Hence, the systems presented in this work address the well-known trade-off between high catalytic activity and stability. Overall, the performance of the surfactant catalyst is better, and in case one of the substrates is hydrophobic, the amphiphilic character proved to be vital. Thus, in future studies it could be worth to change amphiphilicity by the adjustment of the hydrocarbon chain length.

## Methods

All chemicals were used as received, unless otherwise stated. *N,N-*dimethylacetamide (DMA; 99%), acetic acid (100%), cyclen (95%), glycyl-*L*-serine (gly-ser; 97%) and anhydrous cerium(III)-chloride (CeCl_3_; 99.9%) were purchased from *abcr*. Anhydrous scandium(III)-chloride (ScCl_3_; 99.9%), anhydrous ytterbium(III)-chloride (YbCl_3_; 99.9%), *tert-*butyl bromoacetate (98%), trifluoroacetic acid (TFA; 99.9%), benzaldehyde (> 99%), sodium acetate (> 99%), *S*-(11-bromoundecyl) thioacetate (> 90%), indole (> 99%), methyl vinyl ketone (99%), 1-(trimethylsiloxy)cyclohexene (99%) were purchased from *Sigma-Aldrich (Merck)*. BSA protein (in ultrapure 0.9% saline solution with 0.05% sodium azide) was purchased from *Thermo Fisher Scientific)*. Anhydrous zinc(II)-chloride (ZnCl_2_; > 98%) was purchased from *ACROS Organics*. MilliQ-H_2_O was generated using a MilliQ-system. Methanol (MeOH), ethanol (EtOH), tetrahydrofuran (THF), chloroform (CHCl_3_) and dichloromethane (DCM) were used in *p. a.* grade.

### Synthesis

1,4,7-tris(*tert*-butoxycarbonylmethyl)-1,4,7,10-tetraazacyclodode-cane free base (DO3A-^*t*^Bu (3)). As first step, commercially available cyclen (**1**) was selectively tris-alkylated with *tert-*butyl bromoacetate to yield the hydrobromide salt of 1,4,7-*tris*(*tert*-butoxycarbonylmethyl)-1,4,7,10-tetraazacyclododecane (DO3A-^*t*^Bu·HBr (**2**)). In a second step, the corresponding free base was afforded in excellent yields. The synthesis method of Jagadish et al*.*^18^ was reproduced leading to high yields of the tris-alkylated compound (**2**) owing to kinetic control during the nucleophilic substitution reaction at low temperatures. In brief, 5.00 g cyclen (29 mmol, 1.0 eq.) and 7.87 g sodium acetate (96 mmol, 3.3 eq.) were dissolved in 60 mL N*,N*-dimethylacetamide (DMA) under vigorous stirring. 18.7 g *tert-*butyl bromoacetate (14.1 mL, 96 mmol, 3.3 eq.) in 20 mL DMA were added dropwise to the suspension at -20 °C. After vigorous stirring for 24 h, the reaction mixture was poured into 300 mL deionized water and 15 g KHCO_3_ were added. The precipitate was collected by filtration, redissolved in 250 mL CHCl_3_, washed with 100 mL deionized water, dried (MgSO_4_) and concentrated to 25 mL. For the crystallization of (**2**), 250 mL Et_2_O were added. After filtration, washing with Et_2_O and drying *in vacuo*, 5.00 g of (**2**) (8.4 mmol, 1.0 eq.) were dissolved in 250 mL deionized water at 80 °C. 9.4 mL 10% aq. KOH (16.8 mmol, 2.0 eq.) were added at 40 °C and the reaction mixture was stirred for 30 min. After extraction with hexanes (3 × 100 mL), the organic phase was washed with deionized water (3 × 100 mL). The free base (**3**) was yielded as a colorless, viscous oil after a drying step (MgSO_4_), filtration and concentration under reduced pressure. Overall yield **of DO3A-**^***t***^**Bu (3):** 63%. ^1^H-NMR (400 MHz, CDCl_3_): δ 1.44 (br s, 9 H), 1.45 (br s, 18 H), 2.55–2.89 (m, 16 H), 3.31 (s, 2 H), 3.33 (s, 4 H). ESI–MS ( +): 515.376 ([M + H]^+^, calcd. 515.380).

4-(4,7,10-tris(2-(*tert*-butoxy)-2-oxoethyl)-1,4,7,10-tetraazacyclodo-decan-1-yl)butane-1-sulfonate (DO3A-^*t*^Bu-C_4_-SO_3_ (4)). In a typical synthesis, 1.60 g (**3**) (3.1 mmol; 1.0 eq.) were dissolved in 20 mL anhydrous THF under nitrogen gas atmosphere. 0.45 g freshly distilled 1,4-butane sultone (3.3 mmol; 1.05 eq.) were dissolved in 20 mL anhydrous THF and added to the boiling reaction mixture over a period of 20 min. After refluxing for 48 h at 60 °C, the solvent was removed *in vacuo*. A recrystallization step using a mixture of 1/30 MeOH/Et_2_O was performed. The formed colorless precipitate was collected using a filtration step and washed with Et_2_O (3 × 20 mL). After drying *in vacuo*, (**4**) was afforded as a colorless solid. Yield of DO3A**-**^*t*^Bu-C_4_-SO_3_ (4): 76%. Characterization was performed via NMR spectroscopy (^1^H, ^13^C, ^15^ N, COSY & HSQC), via ESI–MS and ATR-IR spectroscopy.

2,2′,2′'-(10-(4-sulfobutyl)-1,4,7,10-tetraazacyclododecane-1,4,7-tri-yl)tri-acetic acid (TFA salt) (DO3A-C_4_-SO_3_ (5)). In a typical TFA deprotection, 1.53 g (**4**) were dissolved in 20 mL TFA under N_2_. The solution was stirred vigorously for 24 h at 25 °C. Remaining TFA was removed *in vacuo* and 2 mL MeOH *p. a.* were added to redissolve the obtained colorless solid residue. At 5 °C, 50 mL Et_2_O were added slowly under stirring. The reaction mixture was stirred for further 2 h without cooling. A filtration step was performed to collect the colorless solid precipitate, which was dried *in vacuo*. Yield of DO3A-C_4_-SO_3_ (5): Quantitative. Characterization of the compound was performed via ^1^H and ^13^C-NMR spectroscopy, via ESI–MS and ATR-IR spectroscopy. ^1^H-NMR (400 MHz, D_2_O): δ 1.76–2.02 (m, 4 H, H_f_), 2.93–3.02 (t, 2 H, H_e_), 3.02–3.24 (m, 8 H, H_d_), 3.29–3.38 (t, 2 H, H_c_), 3.40–3.70 (m, 12 H, H_b_), 4.03–4.15 (s, 2 H, H_a_). ESI–MS ( +): 483.214 ([M + H]^+^, calcd. 483.212); 505.194 ([M + Na]^+^, calcd. 505.194).

Complexation of various transition metal and lanthanide ions (M-DO3A-C_4_-SO_3_ (6)). The complexation of different metal ions using 7 M ammonia in methanol was achieved under strictly anhydrous reaction conditions and inert gas atmosphere (N_2_) to prevent precipitation of insoluble metal hydroxides or oxides. In a typical synthesis, 1.0 eq. TFA salt of (**5**) was dissolved in 10 mL 7 M NH_3_ in MeOH under N_2_. 1.0 eq. of an anhydrous metal chloride salt (MCl_x_) dissolved in 5 mL dry MeOH was added dropwise (N_2_). Depending on the nature of the metal ion, a color change occurred during the addition, e.g. to purple in the case of Ce(III) and some colorless precipitate was formed. The reaction mixture was heated to reflux for 48 h (N_2_). After removal of the solvent *in vacuo*. 20 mL MilliQ-H_2_O were added, ultrasound was applied for 5 min and a syringe-filtration step was performed to remove insoluble residues. The solvent was removed via freeze-drying. In the case of Ce-DO3A-C_4_-SO_3_ (6i) a slightly yellow solid, for Sc-DO3A-C_4_-SO_3_ (6ii), Zn-DO3A-C_4_-SO_3_ (6iii) and Yb-DO3A-C_4_-SO_3_ (6iv) colorless solids were afforded. Yields for the different metal–organic complexes (6): Quantitative. Characterization of the metal complexes was performed via ^1^H and ^13^C-NMR spectroscopy for the diamagnetic complexes Zn-DO3A-C_4_-SO_3_ and Sc-DO3A-C_4_-SO_3_, besides ESI–MS and ATR-IR spectroscopy. For the paramagnetic complexes Ce-DO3A-C_4_-SO_3_ and Yb-DO3A-C_4_-SO_3_ ESI–MS and ATR-IR spectroscopy were performed. For all complexes, IR spectroscopy indicated the presence of ammonium counter-ions.

Tri-*tert*-butyl 2,2′,2′'-[10-(11-(acetylthio)undecyl)-1,4,7,10-tetraaza-cyclododecane-1,4,7-triyl]triacetate (DO3A-^*t*^Bu-C_11_-SAc (7)). 0.50 g **DO3A-**^***t***^**Bu** (**3**) (1.00 mmol; 1.0 eq.) were dissolved in 20 mL CHCl_3_ under nitrogen gas atmosphere. 0.12 g triethylamine (1.20 mmol; 1.2 eq.) were added dropwise under stirring and N_2_. The solution was heated to reflux and 0.33 g *S*-(11-bromoundecyl) thioacetate (1.07 mmol; 1.1 eq.) dissolved in 10 mL CHCl_3_ were added dropwise. The reaction mixture was refluxed for further 24 h (N_2_). The organic phase was extracted with 20 mL distilled water to remove triethylamine salts and then dried (MgSO_4_). The solvent was removed and the obtained pale yellow solid (**7**) was finally dried *in vacuo*. Yield of **DO3A-**^***t***^**Bu-C**_**11**_**-SAc** (**7**): Quantitative. ^1^H-NMR (400 MHz, CDCl_3_): δ 1.23–1.37 (br m, 18 H), 1.43–1.47 (br s, 27 H), 2.31 (s, 3 H), 2.50–3.23 (br m, 18 H), 3.26–3.46 (br m, 8 H). ESI–MS ( +): 743.530 ([M + H]^+^, calcd. 743.535).

2,2′,2′'-(10-(11-(acetylthio)undecyl)-1,4,7,10-tetraaza-cyclododecane-1,4,7-triyl)triacetic acid (DO3A-C_11_-SAc (8)). In a typical deprotection procedure, 0.63 g DO3A-^*t*^Bu-C11-SAc (7) (0.85 mmol) were dissolved in 10 mL DCM and 20 mL TFA were added at 25 °C under nitrogen gas atmosphere. The reaction mixture was stirred for 2 d at 25 °C (N_2_). A yellow-orange solution was obtained. The solvents were removed under reduced pressure. 1.0 g of a yellow oil was afforded, which was purified via recrystallization. The yellow oil was dissolved in 2 mL MeOH and 50 mL Et_2_O were added slowly at 5 °C. A yellow-brownish precipitate formed and the reaction mixture was stirred for further 4 h at 25 °C. The solvents were decanted and the remaining solid residue was washed several times with Et_2_O. DO3A-C_11_-SAc (8) was afforded after a drying step *in vacuo* as yellow solid. Yield of DO3A-C_11_-SAc (8): 82%. ^1^H-NMR (400 MHz, D_2_O): δ 1.25–1.45 (br m, 14 H), 1.52–1.66 (q, 2 H), 1.68–1.85 (m, 2 H), 2.35–2.43 (s, 3 H), 2.87–2.95 (t, 2 H), 3.03–3.31 (m, 12 H), 3.39–3.67 (m, 10 H), 3.91 (s, 2 H). ESI–MS ( +): 575.380 ([M + H]^+^, calcd. 575.347).

### ***M-DO3A-C***_***11***_***-SH (9)***

In a typical synthesis, 0.34 g TFA salt of **DO3A-C**_**11**_**-SAc** (**8**) (0.5 mmol; 1.0 eq.) were dissolved in 10 mL 7 M ammonia in MeOH under nitrogen gas atmosphere and strictly anhydrous conditions. The reaction mixture was stirred for 15 min (N_2_). Then, 78 mg anhydrous ScCl_3_ (0.52 mmol; 1.05 eq.) dissolved in 5 mL dry MeOH were added dropwise (N_2_). The reaction mixture was heated to reflux for 3 d at 75 °C (N_2_). A syringe-filtration step was used to remove insoluble residues from the obtained turbid reaction mixture. The solvent was removed *in vacuo* and 0.29 g of a colorless solid could be afforded after recrystallization from 1/50 MeOH/Et_2_O at 5 °C. Yield of (**9ii**): Quantitative. The compound **Sc-DO3A-C**_**11**_**-SH** (**9ii**) was characterized via ^1^H- and ^13^C-NMR spectroscopy, DOSY and HSQC experiments in MeOD-d_4_. ESI–MS further revealed a successful complexation of Sc^3+^ and deprotection of the thioacetate moiety. This was also investigated via ATR-IR spectroscopy. Amphiphilic properties were examined via pendant drop tensiometry and the capillary rise method. In another synthesis approach, the complexation of Ce(III) via anhydrous CeCl_3_ could be accomplished. 77 mg **DO3A-C**_**11**_**-SAc** (**8**) (0.13 mmol; 1.0 eq.) were dissolved in 10 mL 7 M ammonia in MeOH under N_2_ and strictly anhydrous conditions. The solution was stirred for 10 min at 25 °C. Afterwards, 38 mg anhydrous CeCl_3_ (0.14 mmol; 1.1 eq.) dissolved in 2 mL dry MeOH were added dropwise (N_2_). The reaction mixture was heated to reflux for 2 d (N_2_). A weakly turbid solution was afforded. Yield of (**9i**): Quantitative. Compound **Ce-DO3A-C**_**11**_**-SH** (**9i**) was characterized via ESI–MS.

### Catalytic test reactions.

#### Mukaiyama aldol reaction

30 mg benzaldehyde (0.3 mmol; 1.0 eq.) were suspended in 10.0 g distilled water and a certain amount of catalyst (10 mol-%) was added to the turbid mixture. Then, 53 mg 1-(trimethylsiloxy) cyclohexene (0.31 mmol; 1.1 eq.) were added. The reaction mixture was stirred for 20 h at 25 °C. The organic solvent was removed under reduced pressure and 5.0 mL distilled water were added. The aqueous phase was extracted with 20 mL DCM and after removal of the organic solvent *in vacuo*, the crude reaction product was dried *in vacuo*. Characterization of the obtained colorless viscous liquid was performed via ^1^H-NMR spectroscopy in CDCl_3_. For the calculation of the respective yields of 2-[hydroxy(phenyl)methyl] cyclohexanone, residual signals of benzaldehyde were quantified and subtracted to calculate the yield of the desired aldol reaction product.

#### Michael addition

117 mg indole (1.0 mmol; 1.0 eq.) were suspended in 2.0 g distilled water. A certain amount of catalyst (10 mol-%) was added to the suspension. After 5 min in an ultrasonic bath, 70 mg methyl vinyl ketone (1.0 mmol; 1.0 eq.) were added at 25 °C. After vigorous stirring for 24 h at 25 °C, 2.0 g distilled water were added to the reaction mixture which was subsequently extracted using 10 mL ethyl acetate. The organic phase was dried (MgSO_4_) and the solvent was removed under reduced pressure. After drying, the obtained products were analyzed via ^1^H-NMR spectroscopy in dmso-d_6_. In the case of Sc-DO3A-C_11_-SH (9ii), 117 mg indole (1.0 mmol; 1.0 eq.) were suspended in 4.0 g distilled water and 5.7 mg catalyst (0.01 mmol; 1 mol-%) were dissolved in the turbid mixture. A characteristically smelling yellow oil could be yielded that was further characterized via ^1^H-NMR spectroscopy in dmso-d_6_.

### Hydrolytic cleavage of gly-ser dipeptides

To assess the catalytic activity of the metal–organic complexes containing Lewis acidic metal ions, a test reaction using gly-ser dipeptides was performed in aqueous solution. As a reference, 5.0 mL of a 2 mM solution of dipeptide in distilled water was prepared and stirred for 24 h at 70 °C.

For the paramagnetic compound Ce-DO3A-C_4_-SO_3_ (6i), an equimolar amount of catalyst dissolved in 5.0 mL MilliQ-H_2_O was added to 5.0 mL of a 2 mM aqueous solution of gly-ser dipeptide. ESI–MS (in H_2_O, positive mode) was performed for a qualitative estimation of the afforded cleaved amino acids. For the diamagnetic compounds Sc-DO3A-C_4_-SO_3_ (6ii) and Zn-DO3A-C_4_-SO_3_ (6iii), the reactions were directly conducted in D_2_O. A 5 mM stock solution of gly-ser dipeptide in D_2_O was prepared. The catalysts (5 mM) were added to this solution. The pD was adjusted with diluted NaOD solution. For the calculation of the corresponding pH value, the correction pD = pH + 0.4 was applied. A total volume of 2.0 mL was chosen for each sample. After stirring for 24 h at 70 °C, the pD (resp. pH) was checked again and has remained unchanged. Characterization of the reaction products was performed via ^1^H-NMR spectroscopy. In the case of Sc-DO3A-C_11_-SH (9ii), an equimolar amount of catalyst dissolved in 5.0 mL MilliQ-H_2_O was added to 5 mL of a 2 mM aqueous solution of gly-ser dipeptide. ESI–MS analysis (in H_2_O, positive mode) revealed that the catalyst remained intact. For a quantification of the yielded amino acids glycine and serine, ^1^H-NMR spectroscopy in D_2_O after removal of the solvent *in vacuo* and drying of the obtained solid was performed.

### Cleavage of BSA proteins

A certain amount of surfactant was added to a 2 mM aqueous BSA solution and the pH value was set to 6.0 using diluted NaOH or HCl solution. The reaction mixture was heated to 60 °C and stirred for 16 h. The elevated temperature accelerates the kinetics of the cleavage reaction, e. g. by partial unfolding of the large polypeptide structure. After 16 h of stirring at 60 °C, the reaction mixture was analyzed via SDS-PAGE after it had cooled down to room temperature. Detailed information about the SDS-PAGE analysis are given in the following. The SDS loading buffer solution was composed of a 125 mM Tris–HCl buffer (pH 6.8), 500 mM dithiothreitol (DTT), 0.001 mM bromophenol blue, glycerol, SDS and MilliQ-H_2_O. The SDS running buffer contained 25 mM Tris base, 192 mM glycine and 0.1% SDS (w/v). The stacking gel (4%) was prepared from 1.25 mL 0.5 M Tris–HCl (pH 6.8), 0.65 mL 30% acrylamide monomer, 3.1 mL MilliQ-H_2_O, 35 μL ammonium persulfate (APS), 5 μL *N,N,N′,N′-*tetramethylethylenediamine (TEMED) and 20 μL SDS. The resolving gel (12.5%) was composed of 2.5 mL 1.5 M Tris–HCl (pH 6.8), 4.3 mL 30% acrylamide, 3.3 mL MilliQ-H_2_O, 75 μL APS, 10 μL TEMED and 100 μL SDS. The Coomassie stain solution was prepared using a composition of 0.1% Coomassie Brilliant Blue, 40% EtOH *p. a.*, 10% acetic acid glacial and 49.9% MilliQ-H_2_O. The destaining solution consisted of 40% EtOH, 10% acetic acid glacial and 50% MilliQ-H_2_O. For highly sensitive staining of lower concentrations of polypeptides, a *Krypton* fluorescent protein stain was used. The excitation emission maximum is 520 nm, the emission maximum is 580 nm. The stain is compatible with excitation light sources of 532 nm wavelength for in-gel detection. The *Krypton* stain was commercially available from *Thermo Fisher Scientific*. Depending on the amount of polypeptide, the time duration of staining was adapted. In brief, the stain (10X stock solution) was diluted ten-fold with MilliQ-H_2_O at 25 °C. Then, having the gels removed from the gel cassette, they were immersed in a tray with sufficient volume of gel fixing solution. After agitating on a shaker for 30 min, the fixing solution was decanted and MilliQ-H_2_O was added carefully to remove residual solution from the gel. Again, the water was decanted and afterwards, the gel was immersed in a sufficient amount of 1X *Krypton* protein stain and covered using a piece of aluminum foil. The tray was placed on a shaker and stained overnight under smooth agitation. Finally, the stain was decanted and the destaining solution was added. The tray was covered and agitated for further 5 min. The destaining solution was decanted and MilliQ-H_2_O was added. After agitation for 15 min, the water was decanted. This process was repeated two times with increasing volumes of MilliQ-H_2_O. For the detection of the bands, the gel can be imaged on any platform equipped with appropriate excitation and emission filters. Values are all given in v/v, unless otherwise stated. The unstained protein standard was commercially available from BioLabs and exhibited a band profile of 10, 15, 25, 30, 40, 50, 60, 70, 85, 100, 120, 150 and 200 kDa. For the protein analysis via SDS-PAGE, 12.5% polyacrylamide gels were freshly prepared. Each of the investigated samples was mixed with an equal volume of SDS loading buffer (typically 10 μL) and was afterwards heated to 95 °C for 5–10 min. Then, after the solutions had come to room temperature, the samples were loaded into the wells. The electrodes were connected to perform the electrophoresis at a constant current of 35 mA. After 25 min, the electrophoresis was finished and the gel was incubated overnight with an appropriate staining solution. The destain solution was added to the gel after the staining process with subsequent rinsing with MilliQ-H_2_O (see above for the exact experimental procedure). After destaining, the gels were stored in a gel storage solution and photographed.

### Analytical methods

400 MHz ^1^H-NMR and 100 MHz ^13^C-NMR spectra were recorded on a *Bruker Avance III 400* at 298 K. 600 MHz ^1^H-NMR and 125 MHz ^13^C-NMR spectra were recorded on a *Bruker Avance III 600* at 298 K. 800 MHz ^1^H-NMR and 200 MHz ^13^C-NMR spectra were recorded on a *Bruker Avance Neo 800* at 298 K. ESI–MS analysis was performed on a *Bruker Microtof II* system. The samples were directly injected into the evaporation chamber after syringe filtration using an appropriate solvent. ATR-FT-IR spectra for the investigation of dried solid samples were conducted on a *Perkin Elmer Spectrum 100* device using a Perkin Elmer Universal ATR sampling accessory. DLS was measured on a *Malvern Zetasizer μV* at 90° detection angle. UV–VIS measurements were performed on a *Thermo Scientific Genesys* spectrophotometer using a wavelength range between 190 and 800 nm.

Pendant drop measurements for the determination of the surface tension were performed using *KRÜSS* ADVANCE *1.8.0.4* software. As second independent method for the measurement of concentration dependent surface tension, commercially available glass capillaries were used in the capillary rise method. TEM was conducted on a *Zeiss Libra 120* microscope (120 kV) and HR-TEM on a *JEOL JEM-2200FS* microscope at 200 kV acceleration voltage. The samples were placed on carbon-coated copper grids (mesh 400).

## Supplementary Information

Supplementary Information
