# Supplementary material for: Molecular fusion of surfactant and Lewis-acid properties for attacking dirt by catalytic bond cleavage
Source: Sci Rep. 2021 Mar 4;11:5131. doi: 10.1038/s41598-021-84654-3 (PMC7933239; doi:10.1038/s41598-021-84654-3)
Supplement: Supplementary file 1 — Supplementary Information [file 41598_2021_84654_MOESM1_ESM.pdf]

Molecular Fusion of Surfactant and Lewis-Acid Properties for Attacking Dirt by Catalytic Bond Cleavage

Marvin L. Frisch[b,c] and Sebastian Polarz*[a,b]

[a] Prof. Dr. S. Polarz, Institute for Inorganic Chemistry, Leibniz-University Hannover, Callinstrasse 9, 30167 Hannover, Germany

[b] M. Frisch, Prof. Dr. S. Polarz, Department of Chemistry, University of Konstanz, Universitaetsstrasse 10, 78457 Konstanz, Germany

[c] M. Frisch, Institute of Chemistry, Technical University Berlin, Strasse des 17. Juni 124, 10623 Berlin, Germany

SUPPORTING INFORMATION

Fig. SI-1. Analysis of the nucleophilic ring-opening reaction of DO3A-^{t}Bu (**3**) with 1,4-butane sultone for the synthesis of DO3A-^{t}Bu-C_4-SO_3 (**4**)

(a) NMR spectroscopic methods

^{1}H- (left) and ^{15}N-NMR (right) spectra of (**4**):

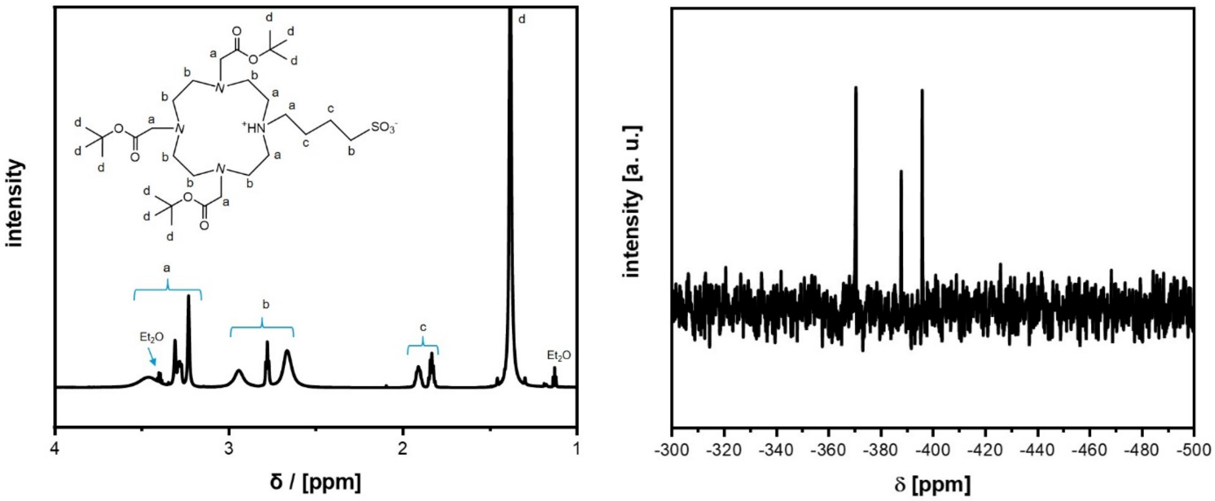

^{1}H-NMR (800 MHz, $CDCl_3$): δ 1.38 (br s, 27 H, H_d), 1.80 – 1.94 (m, 4 H, H_c), 2.55 – 3.02 (m, 14 H, H_b), 3.18 – 3.55 (m, 12 H, H_a).

^{15}H-NMR (800 MHz, $CDCl_3$): δ -370.7, -388.1, -396.2 ppm (ref. to $MeNO_2$).

In the ^{1}H-NMR spectrum of compound (**4**), line-broadening effects of the methylene C**H**$_2$ signals (H_b) become evident. This can be explained by the inhibition of their rotational degrees of freedom due to the cyclic nature of the macrocycle.

DOSY of (**4**):

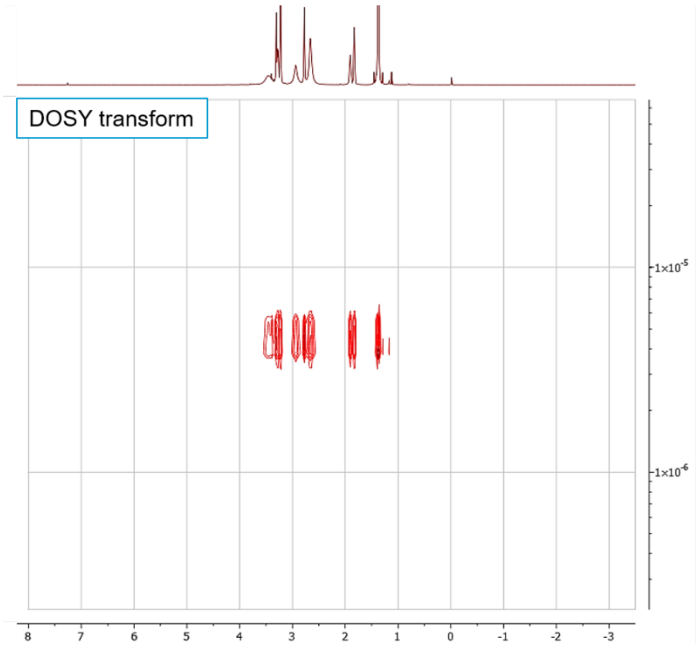

DOSY was acquired at 800 MHz at a concentration of 1 mol/L of (**4**) in $CDCl_3$. The x-axis corresponds to the chemical shift in ppm and the y-axis corresponds to the logarithm of the diffusion coefficient in cm^2/s. A diffusion coefficient of $4.51 \cdot 10^{-10}$ m^2/s was calculated. According to the Stokes-Einstein equation, this equals a hydrodynamic diameter of 1.8 nm, which is in accordance with the molecular structure and dimension of the compound.

(b) ESI-MS

ESI-MS pattern of (**4**) in negative mode:

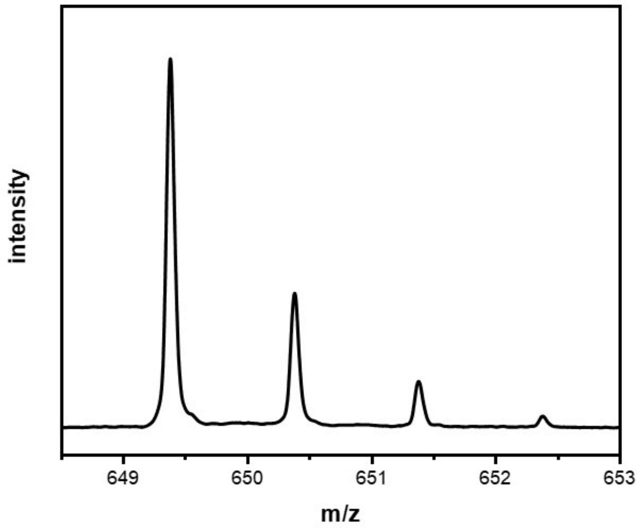

ESI-MS (-): 649.382 ($[M]^-$, calcd. 649.385).

(c) IR spectroscopy

ATR-IR spectrum of (**4**):

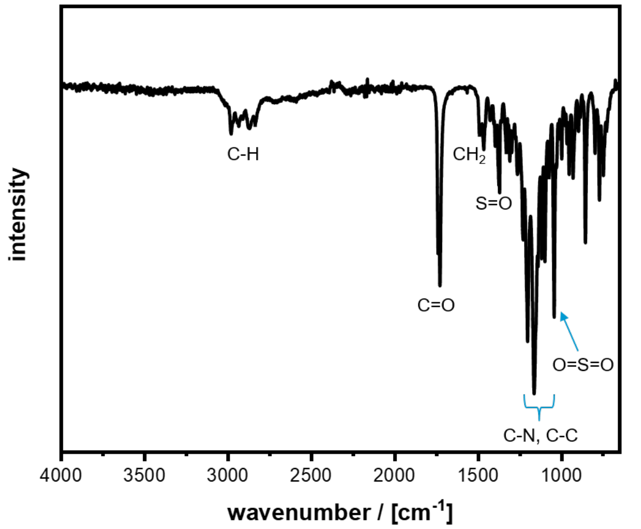

Fig. SI-2. Characterization of the organic ligand (**5**)

(a) NMR spectroscopy

^{1}H-NMR spectrum of (**5**):

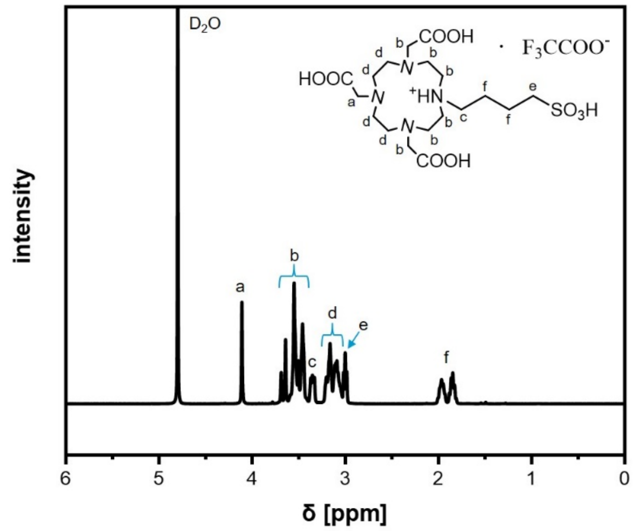

^{1}H-NMR (400 MHz, D_2O): δ 1.76 – 2.02 (m, 4 H, H_f), 2.93 – 3.02 (t, 2 H, H_e), 3.02 – 3.24 (m, 8 H, H_d), 3.29 – 3.38 (t, 2 H, H_c), 3.40 – 3.70 (m, 12 H, H_b), 4.03 – 4.15 (s, 2 H, H_a).

(b) ESI-MS

ESI-MS pattern of (**5**) in positive mode:

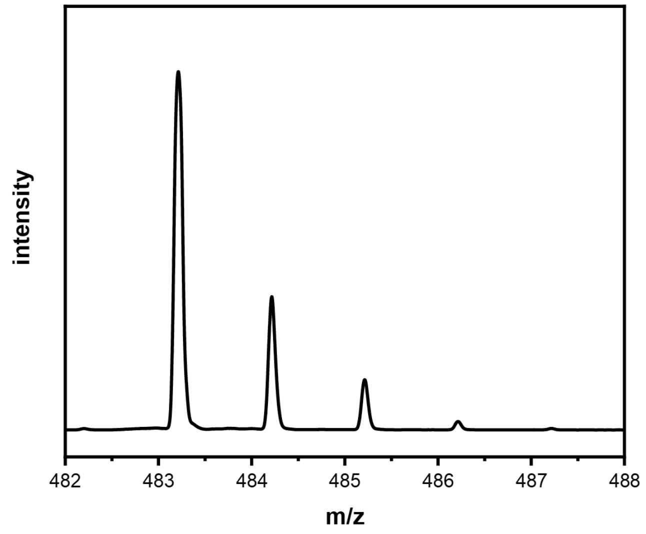

ESI-MS (+): 483.214 ($[M+H]^+$, calcd. 483.212); 505.194 ($[M+Na]^+$, calcd. 505.194).

(c) IR spectroscopy

ATR-IR spectrum of (**5**):

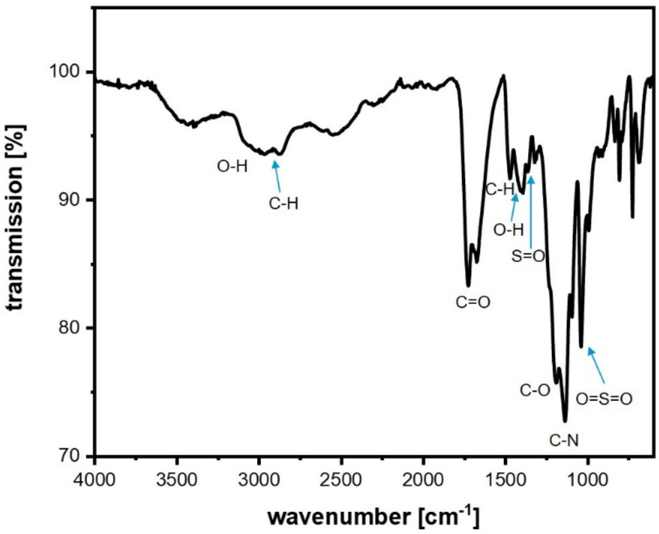

IR spectroscopy confirms the covalent attachment of the C_4 alkyl side chain terminated by a sulfonate end group to the tris-alkylated cyclen moiety prior to and after the removal of the ^{t}Bu protecting groups using TFA. The presence of O-H vibrations very likely occurs due to the hygroscopic nature of the organic ligand.

Fig. SI-3. Characterization of M-DO3A-C_4-SO_3 (**6**)

(a) NMR spectroscopy

^{1}H-NMR spectrum of **Zn-DO3A-C_4-SO_3**:

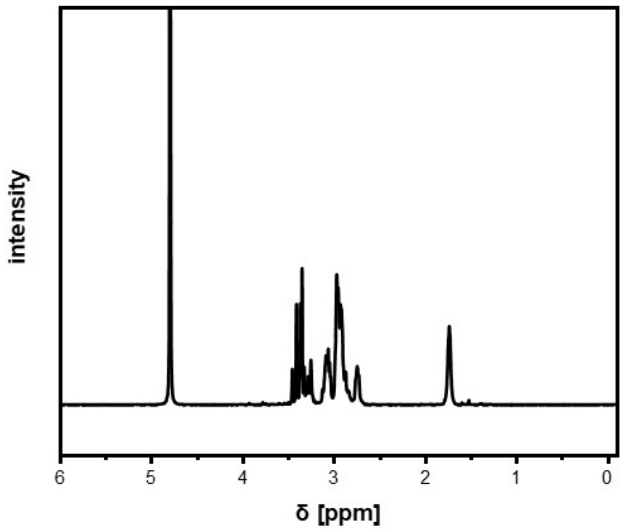

^{1}H-NMR (400 MHz, D_2O): δ 1.64 – 1.81 (br s, 4 H), 2.70 – 3.11 (m, 18 H), 3.20 – 3.48 (m, 8 H).

^{13}C-NMR (100 MHz, D_2O): δ 21.7, 22.1, 50.7, 51.0, 52.3, 54.6, 54.9, 56.7, 58.0, 60.2, 176.4, 178.7.

Low-field region of ^{13}C-NMR spectra of the organic ligand (**5**) and Zn-DO3A-C_4SO_3:

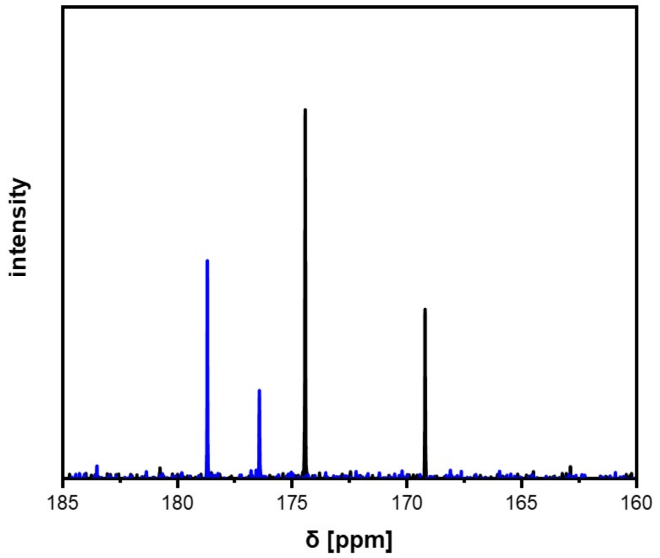

By ^{13}C-NMR spectroscopy, an indication for a strong metal-ligand coordination by the carboxylic acid moieties of the macrocyclic ligand can be made. This becomes clear in the low-field region of the ^{13}C-NMR spectra of Zn-DO3A-C_4-SO_3 (blue) and DO3A-C_4-SO_3 (**5**) (black) and is consistent with previous findings from Hermann *et al.*[1] The observed downfield-shift of the signals stemming from the carboxylic acid carbon atoms can be explained by the complexation of positively charged metal ions exerting an electron-withdrawing effect.

^1H-NMR spectrum of **Sc-DO3A-C_4-SO_3**:

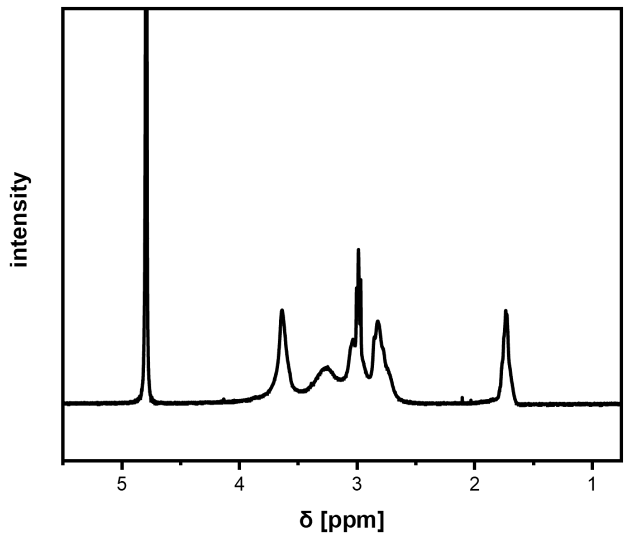

^1H-NMR (400 MHz, D_2O): δ 1.60 – 1.83 (br s, 4 H), 2.63 – 3.42 (m, 20 H), 3.50 – 3.77 (m, 6 H).

^{13}C-NMR (100 MHz, D_2O): δ 18.8, 22.2, 50.6, 54.0, 54.9, 55.9, 59.3, 65.7, 66.5, 180.6.

(b) ESI-MS

ESI-MS pattern of **Zn-DO3A-C_4-SO_3** in negative mode:

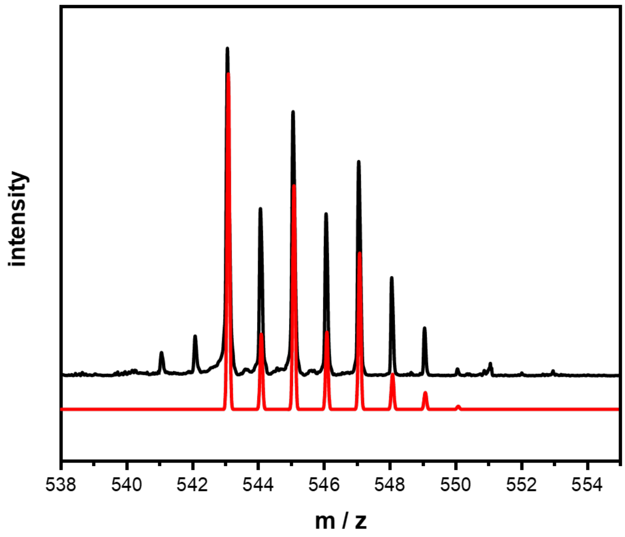

ESI-MS (-): 543.089 ($[M-H]^-$, calcd. 543.111)

ESI-MS of **Sc-DO3A-C_4-SO_3**:

ESI-MS (+): 1049.230 ($[2M+H]^+$, calcd. 1049.280); 1071.210 ($[2M+Na]^+$, calcd. 1071.263).

ESI-MS of **Ce-DO3A-C_4-SO_3**:

ESI-MS (+): 620.100 ($[M+H]^+$, calcd. 620.094); 642.079 ($[M+Na]^+$, calcd. 642.076).

ESI-MS of **Yb-DO3A-C_4-SO_3**:

ESI-MS (+): 654.124 ($[M+H]^+$, calcd. 654.126); 671.151 ($[M+NH_4]^+$, calcd. 671.154).

(c) IR spectroscopy

Another powerful method for the investigation of the metal-ligand coordination is ATR-IR spectroscopy. The carbonyl C=O stretch vibration of all metal-organic complexes LA-DO3A-C_4-SO_3 is shifted towards lower wavenumbers compared to that in the organic ligand (**5**). On the other hand, the C-O stretch vibration is shifted to higher wavenumbers. The reason for these shifts of the C=O and C-O stretches to lower or higher energies, respectively, lies in a transfer of electron density onto the metal center ion *via* complexation by the chelating ligand and was previously explained by Hermann *et al.*[1]

Wavenumbers for the stretch vibrations of C-O and C=O bonds:

	**C-O [cm^{-1}]**	**C=O [cm^{-1}]**
DO3A-C_4-SO_3 (**5**)	1185	1668
Zn-DO3A-C_4-$SO_3(NH_4)_2$	1217	1586
Ce-DO3A-C_4-SO_3NH_4	1200	1575
Sc-DO3A-C_4-SO_3NH_4	1201	1591
Yb-DO3A-C_4-SO_3NH_4	1199	1584

For all investigated compounds, O=S=O stretching vibrations were found at about 1050 cm^{-1}. This is further proof for the terminating sulfonate group.

Fig. SI-4. Characterization of DO3A-C_{11}-SAc (**8**)

Compound (**8**) was afforded *via* TFA-catalyzed cleavage of (**7**) (see Experimental Section).

(a) NMR spectroscopy

^{1}H-NMR spectrum of (**8**):

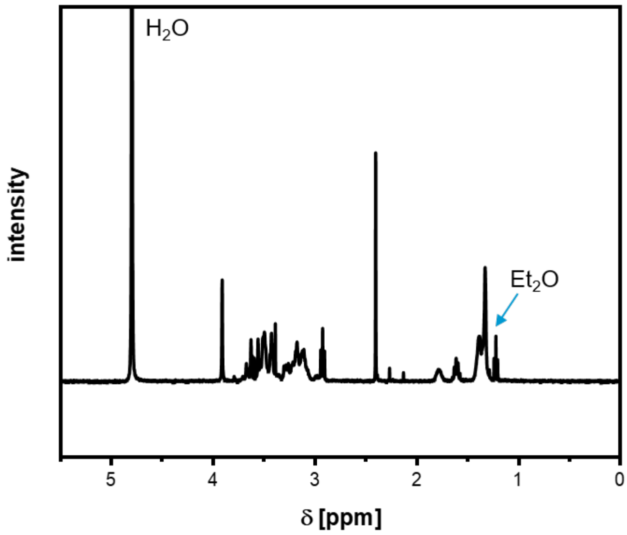

^{1}H-NMR (400 MHz, D_2O): δ 1.25 – 1.45 (br m, 14 H), 1.52 – 1.66 (q, 2 H), 1.68 – 1.85 (m, 2 H), 2.35 – 2.43 (s, 3 H), 2.87 – 2.95 (t, 2 H), 3.03 – 3.31 (m, 12 H), 3.39 – 3.67 (m, 10 H), 3.91 (s, 2 H).

(b) ESI-MS

ESI-MS pattern of (**8**) in positive mode:

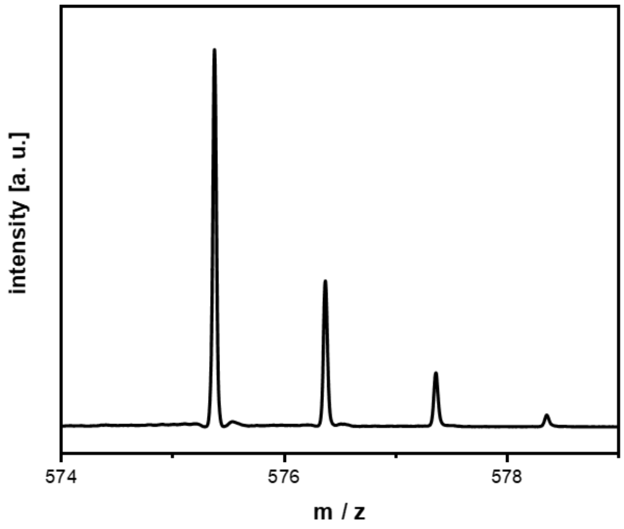

ESI-MS (+): 575.380 ($[M+H]^+$, calcd. 575.347).

Fig. SI-5. Characterization of Sc-DO3A-C_{11}-SH (**9ii**)

(a) NMR spectroscopy

^{1}H-NMR spectrum of (**9ii**):

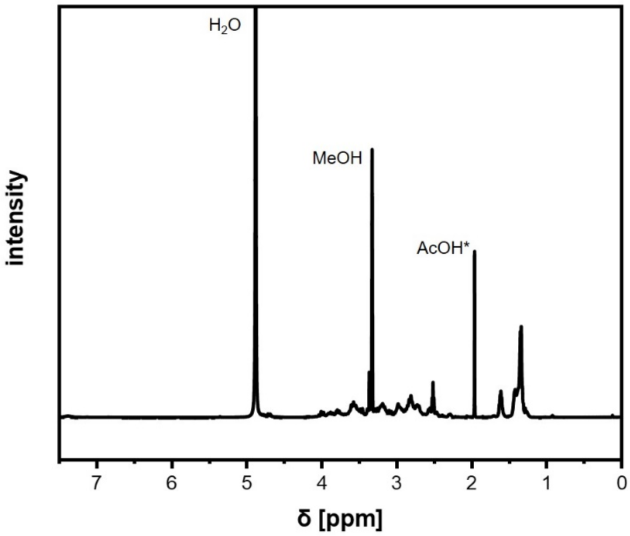

^{1}H-NMR (600 MHz, MeOH-d_4): δ 1.20 – 1.70 (m, 18 H), 2.42 – 3.26 (m, 18 H), 3.38 – 4.00 (m, 8 H).

The three sharp signals indexed in the spectrum can be attributed to signals from the solvent in the NMR experiment. This becomes more evident in the acquired DOSY spectrum shown below.

^{13}C-NMR (125 MHz, MeOH-d_4): δ 18.9, 19.0, 22.8, 26.6, 26.9, 27.2, 28.0, 28.5, 28.6, 50.6, 53.5, 54.2, 55.3, 64.6, 65.1, 65.3, 65.6, 65.9, 66.2, 174.2, 177.6.

DOSY of (**9ii**):

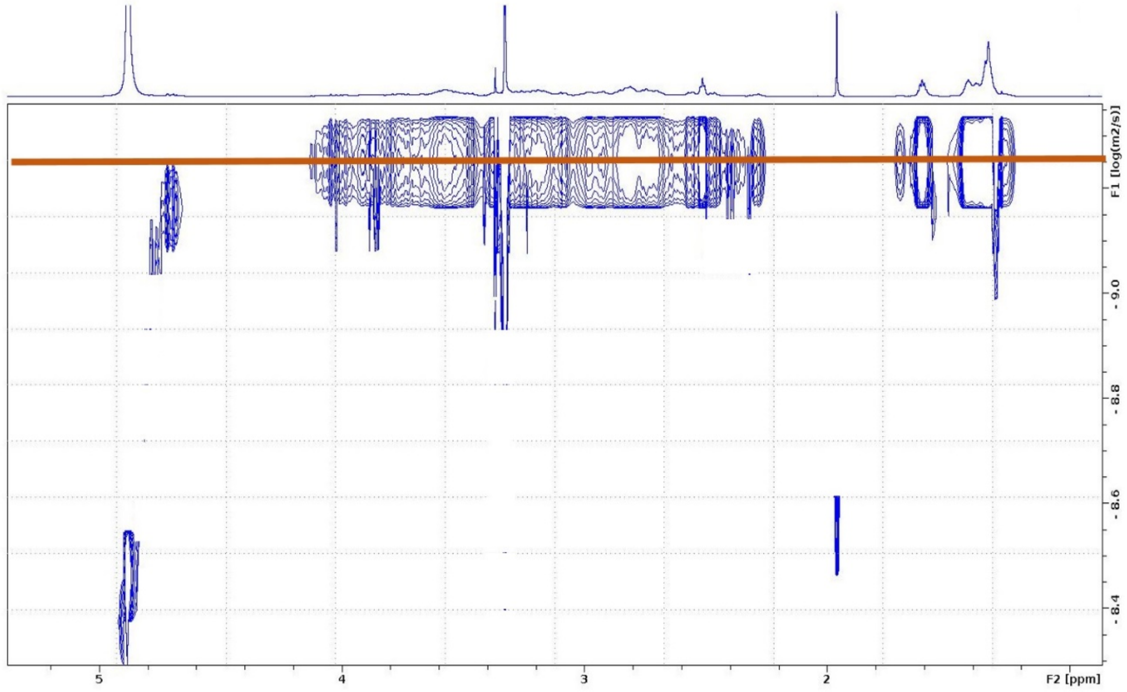

DOSY was acquired at 800 MHz methanol-d_4 and T = 25 °C. The x-axis corresponds to the chemical shift in ppm and the y-axis corresponds to the logarithm of the diffusion coefficient in m^2/s. There are three distinguishable species according to the evaluation and fitting of the DOSY raw data. Two of them can be assigned to the solvent. The third species, which is marked by the orange horizontal bar, corresponds to Sc-DO3A-C_{11}-SH (**9ii**). Using to the Stokes-Einstein equation, a hydrodynamic diameter of 1.5 nm was calculated. This value is smaller than expected, considering the eleven CH_2 units in the hydrophobic alkyl chain. For a fully extended alkyl chain terminated by a thiol group, (**9ii**) is expected to have a hydrodynamic diameter of more than 2 nm. Possibly, the alkyl chain is partially back-folded in the methanolic solution, decreasing the calculated value for D_h. In the DOSY spectrum some of the signals are strongly broadened (vertical tailing). This might be an indication of different structural conformations of a long and flexible alkyl chain. Besides, DOSY analysis confirms the high purity of the synthesized compound (**9ii**).

(b) IR spectroscopy

Wavenumbers for the stretch vibrations of C-O and C=O bonds:

	**C-O [cm^{-1}]**	**C=O [cm^{-1}]**
Sc-DO3A-C_{11}-SH	1217	1586

ATR-IR spectrum of (**9ii**):

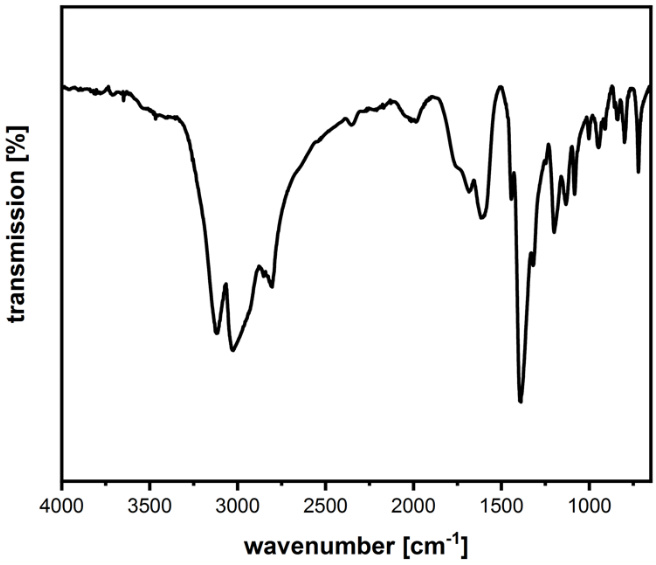

Compared to the IR spectra of M-DO3A-C_4-SO_3 compounds, the IR spectrum of (**9ii**) shows no signs of N-H vibrations. This can be explained by the less acidic alkyl –SH group compared to the highly acidic –SO_3H group of the C_4 complexes. A weak S-H absorption band is visible at about 2550 cm^{-1}.[2]

Fig. SI-6. Stability tests of Sc-DO3A-C_{11}-SH (**9ii**) in aqueous solutions

Photograph of a solution of (**9ii**) after 48 h at pH 12:

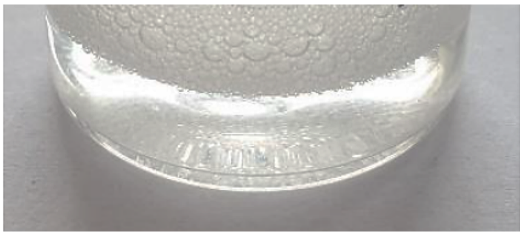

To assess the hydrolytic stability of M-DO3A-C_{11}-SH compounds, (**9ii**) was dissolved at a concentration of 1 mg/mL in water at elevated pH (pH 12). The pH of the solution was adjusted using diluted NaOH solution. As can be seen on the image, there is no precipitate visible by eye. The foam on top of the solution suggests the amphiphilic character of the compound.

Fig. SI-7. TEM images of Sc-DO3A-C_{11}-SH (**9ii**)

TEM image of (**9ii**) showing multilayer sheets *in vacuo* (scale bar = 100 nm):

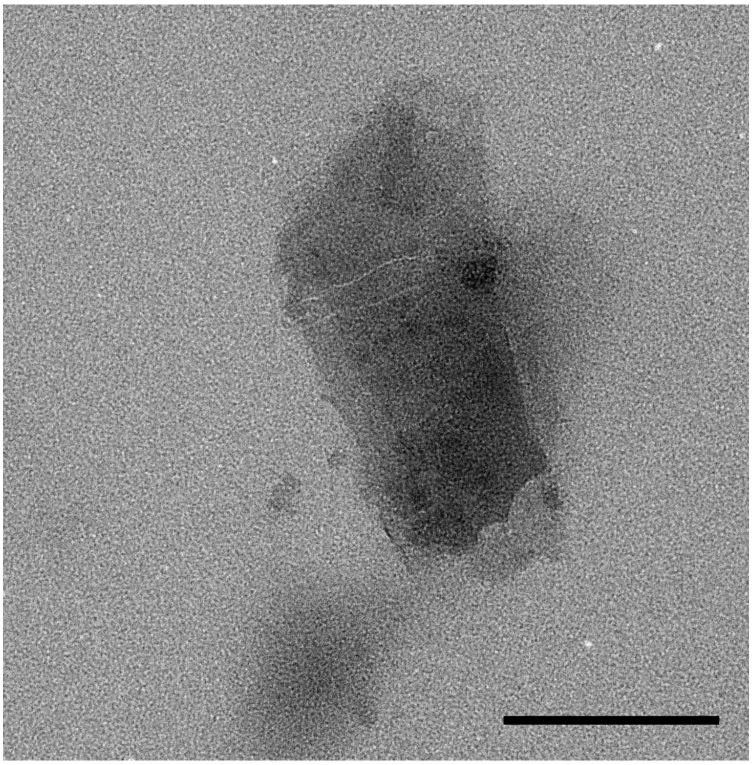

The size of the aggregates observed *via* TEM is about 150 – 200 nm. This is in good agreement with the average particle size determined *via* DLS measurements in aqueous solution. TEM-EDX analysis revealed a ratio of 1.1 to 1 for scandium to sulfur for (**9ii**). Moreover, scandium was homogeneously distributed over the whole particle. No other elements than Sc, S, C, N, O and Cu (from copper grid) were found.

Fig. SI-8. Integrity of the metal-organic complexes after the catalytic test reactions

Representative ESI-MS spectrum of (**9ii**) after the hydrolytic cleavage of gly-ser dipeptides for 24 h at 70 °C and pH 7:

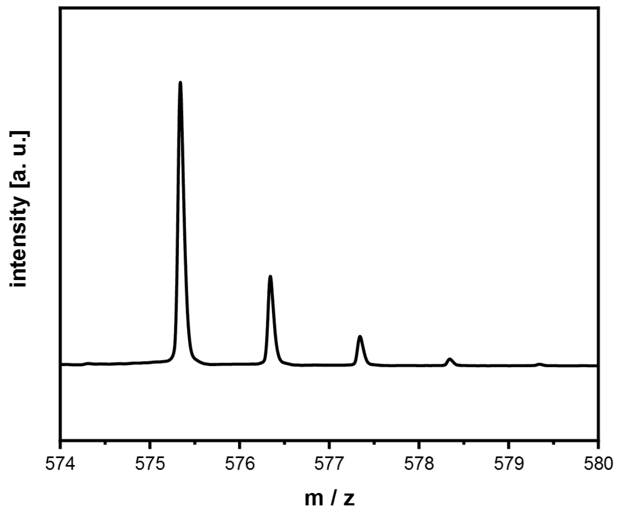

ESI-MS analysis revealed the integrity of the catalysts after the different catalytic test reactions. The above spectrum exemplarily shows the result for the most active compound (**9ii**) in the hydrolytic cleavage of gly-ser dipeptides at elevated temperature. Similar observations were made for the other compounds. It can be concluded that the metal cation is still coordinated by the macrocyclic ligand for compounds (**6**) and (**9**) after the catalytic test reactions.

4. Catalytic Testing

4.1 Bond-Formation Reactions

Table **SI-1**: Calculated yields for the Mukaiyama aldol-type reaction of hydrophobic benzaldehyde with the water-labile silyl enol ether 1-(trimethylsiloxy)cyclohexene:

**catalyst**	**T**	**t**	**amount of catalyst**	**degree of conversion**
-	25 °C	20 h	-	0 %
DO3A-C_4-SO_3	25 °C	20 h	10 mol-%	0 %
$CeCl_3$	25 °C	20 h	10 mol-%	0 %
$ScCl_3$	25 °C	20 h	10 mol-%	0 %
$ZnCl_2$	25 °C	20 h	10 mol-%	0 %
$YbCl_3$	25 °C	20 h	10 mol-%	0 %
Ce-DO3A-C_4-SO_3 (**6i**)	25 °C	20 h	10 mol-%	0 %
Sc-DO3A-C_4-SO_3 (**6ii**)	25 °C	20 h	10 mol-%	0 %
Zn-DO3A-C_4-SO_3 (**6iii**)	25 °C	20 h	10 mol-%	0 %
Yb-DO3A-C_4-SO_3 (**6iv**)	25 °C	20 h	10 mol-%	0 %
Sc-DO3A-C_{11}-SH (**9ii**)	25 °C	20 h	10 mol-%	**30 %**

Yields were calculated *via* ^{1}H-NMR spectroscopy. Without any organic co-solvent or the addition of surfactants, (**9ii**) was the only catalyst to afford any reaction product. For all other examined compounds, including the bare organic ligand, no reaction product was found in the organic phase after extraction using DCM. On the contrary, for (**9ii**), surface activity combined with Lewis acidic properties afforded some aldol-type reaction product in the extracted organic phase. The extracted aldol-type reaction product was analyzed *via* ^{1}H-NMR spectroscopy (400 MHz, $CDCl_3$):

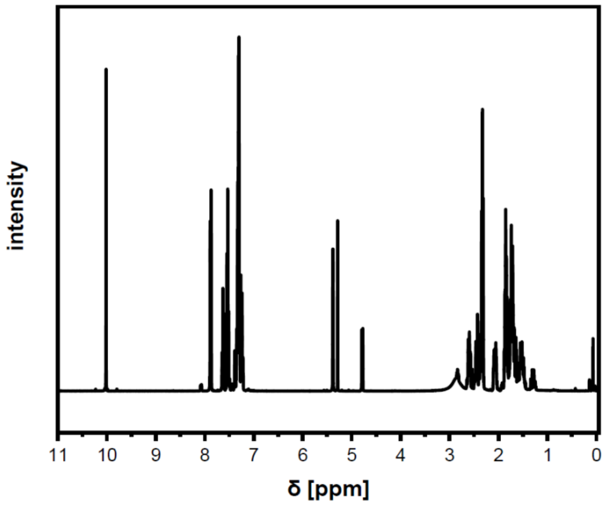

The spectrum reveals the presence of residual benzaldehyde reactant indicated by the aldehyde proton at 10.0 ppm. The amount of the latter was calculated to be about 57 %. Taking the entire mass of afforded reaction products into account, a conversion degree of the desired aldol product of 30 % results for compound (**9ii**).

Table **SI-2**: Calculated yields for the Michael addition of hydrophobic indole with methyl vinyl ketone:

**catalyst**	**T**	**t**	**amount of catalyst**	**degree of conversion**
-	25 °C	24 h	-	4 %
$CeCl_3$	25 °C	24 h	10 mol-%	17 %
$ScCl_3$	25 °C	24 h	10 mol-%	19 %
DO3A-C_4-SO_3	25 °C	24 h	10 mol-%	4 %
DO3A-C_{11}-SH	25 °C	24 h	1 mol-%	5 %
Ce-DO3A-C_4-SO_3 (**6i**)	25 °C	24 h	10 mol-%	5 %
Sc-DO3A-C_4-SO_3 (**6ii**)	25 °C	24 h	10 mol-%	11 %
Sc-DO3A-C_{11}-SH (**9ii**)	25 °C	24 h	**1 mol-%**	26 %

Yields were calculated *via* ^{1}H-NMR spectroscopy after extraction of the crude reaction mixture using ethyl acetate. The extracted Michael addition product was analyzed *via* ^{1}H-NMR spectroscopy (400 MHz, dmso-d_6):

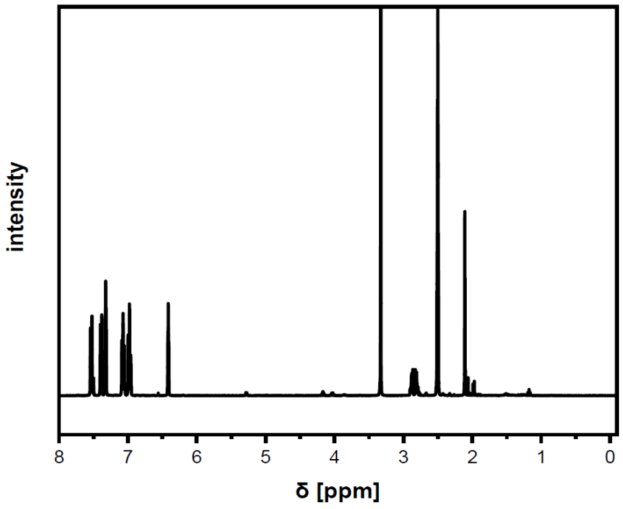

The ^{1}H-NMR spectrum shown above corresponds to the obtained reaction products using (**9ii**) as catalyst. It reveals the presence of unconverted indole indicated by the signal at 6.4 ppm. The latter was used as reference for the calculation of the degree of conversion. The ^{1}H-NMR spectra of the other test reactions using different catalysts look similar and calculations were performed similarly.

4.2 Bond-Cleavage Reactions

4.2.1 Hydrolytic cleavage of gly-ser dipeptides at neutral pH

The hydrolytic cleavage of unactivated amide bonds in glycine-*L*-serine dipeptides was investigated in aqueous medium at neutral pH.

Table **SI-3**: Calculated yields for the cleavage of gly-ser dipeptides at pH 7:

catalyst	T	t	amount of catalyst	yield of glycine + serine	yield of c(gly-ser)
-	70 °C	24 h	-	7 %	0 %
DO3A-C_4-SO_3	70 °C	24 h	equimolar	7 %	0 %
DO3A-C_{11}-SH	70 °C	24 h	equimolar	8 %	0 %
Zn-DO3A-C_4-SO_3 (**6iii**)	70 °C	24 h	equimolar	8 %	0 %
Sc-DO3A-C_4-SO_3 (**6ii**)	70 °C	24 h	equimolar	13 %	6 %
Sc-DO3A-C_{11}-SH (**9ii**)	70 °C	24 h	equimolar	32 %	20 %

Yields were calculated *via* ^{1}H-NMR spectroscopy in D_2O. ^{1}H-NMR spectra revealed that compound (**6iii**) hardly had any catalytic effect on the cleavage of the amide bond in the dipeptide, as the degree of conversion is basically the same as without any catalyst or the metal-free ligand DO3A-C_4-SO_3, where 7 % of the dipeptide were cleaved into the corresponding amino acids. This is in good agreement with literature.[3,4] For compounds (**6ii**) and (**9ii**) the formation of cyclic dipeptide, c-(gly-ser), is observed, as already pointed out by Ly *et al.*[3] who suggested that this would be a reversible process. Importantly, no formation of a gel or any precipitation occurred during the test reaction. This indicates a high stability of the catalysts in the presence of the dipeptides and reaction products in aqueous solution. To point out the positive impact of Lewis-acidity on the catalytic bond cleavage reaction, a reference experiment was conducted with the metal-free ligand DO3A-C_{11}-SH under identical reaction conditions. Yields were significantly lower compared to the metal-containing compound (9ii), highlighting the beneficial effects of Lewis-acidic surfactants on catalytic performance.

As mentioned in the manuscript, the catalytic cleavage of gly-ser dipeptides was also studied for paramagnetic Ce-DO3A-C_4-SO_3 (**6i**). After 3 d of stirring at 60 °C and pH 7, ESI-MS analysis qualitatively confirmed the presence of cleaved amino acids and, hence, the catalytic activity of the compound owing to its Lewis acidic properties. As a reference experiment, (see left image below) the reaction was performed without the addition of any catalyst. ESI-MS analysis of the reaction mixture containing (**6i**) (see right image given below), the amount of free amino acid serine (blue-framed peak corresponding to the sodium adduct $[M+Na]^+$) relative to the dipeptide (grey-framed; $[M+H]^+$ and $[M+Na]^+$) is significantly higher. Spectra were recorded in water and positive mode. Concentrations of the injected solutions were similar.

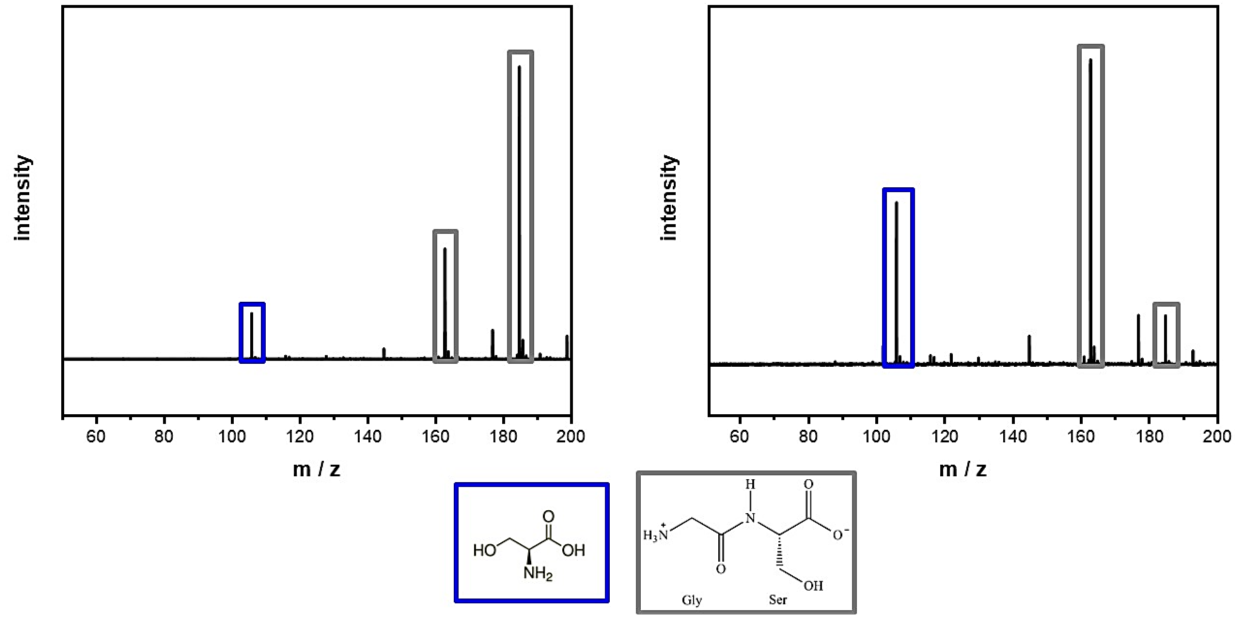

4.2.2 Hydrolytic cleavage of BSA using Ce-DO3A-C_4-SO_3 or $CeCl_3$ pretreated in alkaline solution

To further assess the hydrolytic stability of the synthesized metal complexes, Ce-DO3A-C_4-SO_3 (**6i**) was chosen as representative model system. Prior to catalytic bond fission testing, a 10 mM aqueous solution of (**6i**) was set to pH 10 using diluted sodium hydroxide solution. After stirring for 24 h at 40 °C, no indications for any precipitation of insoluble, colored cerium (oxy)hydroxides were given. The pH of the clear solution remained constant (pH 10). Then, the solvent was removed *via* freeze-drying. The obtained slightly yellow solid powder was finally used for the catalytic bond fission reactions using BSA (results from SDS-PAGE analysis are given in the figure below).

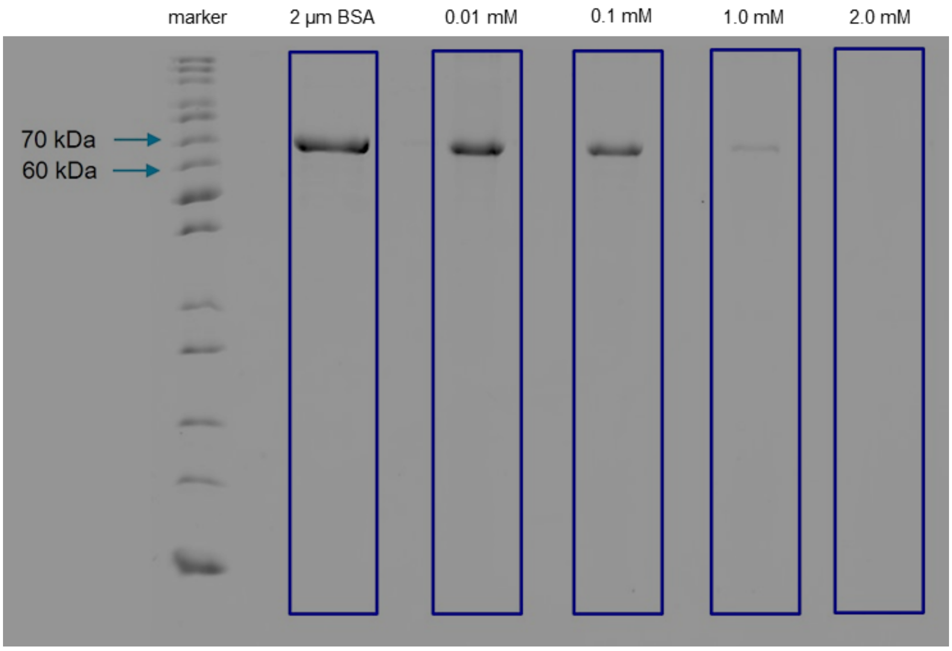

A 1000-fold molar excess of cleavage agent (**6i**) ensures a complete cleavage of BSA after 16 hours at 60 °C (pH 6.0). These results suggest high cleavage activity, comparable to compound (**9ii**). Performing the whole experiment with $CeCl_3$ salt instead of (**6i**), a purple metal (oxy)hydroxide was obtained and used as cleavage agent after drying. A 1000-fold molar excess did not lead to any visible signs for BSA protein cleavage under similar reaction conditions. Hence, the superior ability of bond fission of (**6i**) is clearly evidenced, as no significant amounts of catalytically inactive cerium (oxy)hydroxide species were formed due to the high hydrolytic stability of the complex in alkaline medium.

Fig. SI-9. Full-length gel of SDS-PAGE (original measurement file) of the hydrolytic cleavage of BSA at pH 6 and 60 °C for 16 h using Sc-DO3A-C_{11}-SH (**9ii**) as a surfactant-cleavage agent.

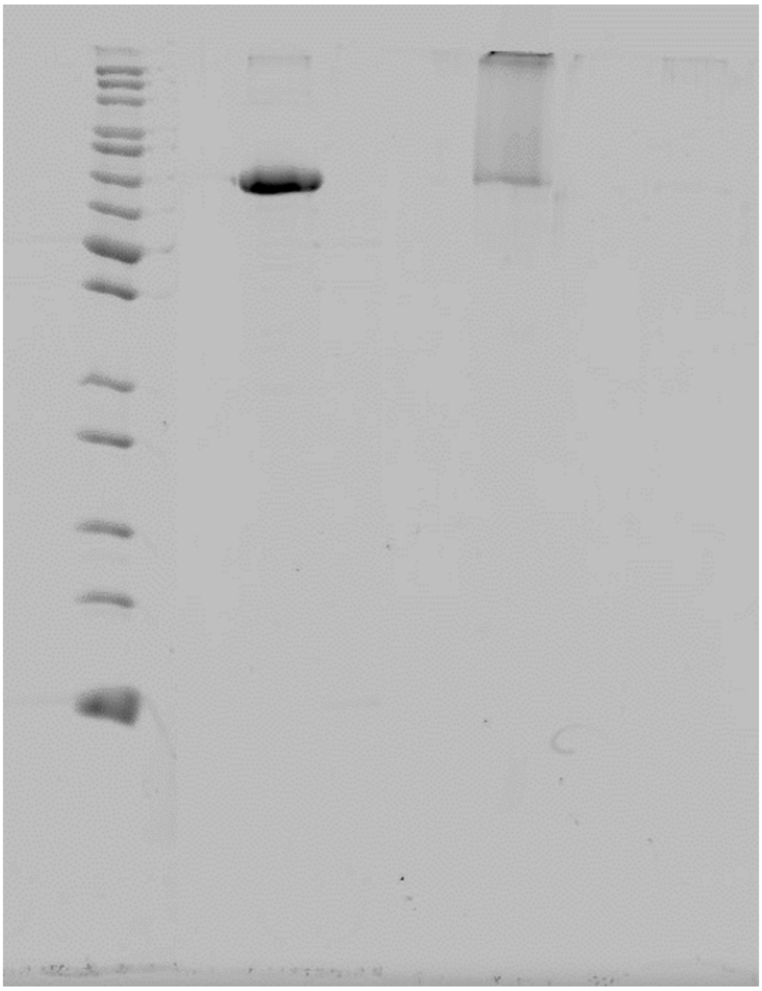

5. References

1 Hermann, S. *et al.* Magneto-Adaptive Surfactants Showing Anti-Curie Behavior and Tunable Surface Tension as Porogens for Mesoporous Particles with 12-Fold Symmetry. *Angew. Chem.-Int. Edit.* **56**, 5475-5479, doi:10.1002/anie.201612416 (2017).

2 Tingaut, P., Hauert, R. & Zimmermann, T. Highly efficient and straightforward functionalization of cellulose films with thiol-ene click chemistry. *Journal of Materials Chemistry* **21**, 16066-16076, doi:10.1039/C1JM11620G (2011).

3 Ly, H. G. T., Absillis, G. & Parac-Vogt, T. N. Amide bond hydrolysis in peptides and cyclic peptides catalyzed by a dimeric Zr(iv)-substituted Keggin type polyoxometalate. *Dalton Transactions* **42**, 10929-10938, doi:10.1039/C3DT50705J (2013).

4 Yashiro, M. *et al.* Metal-ion-assisted hydrolysis of dipeptides involving a serine residue in a neutral aqueous solution. *Organic & Biomolecular Chemistry* **1**, 629-632, doi:10.1039/B209565C (2003).
